# Targeted plasma proteomics reveals upregulation of distinct inflammatory pathways in people living with HIV

**DOI:** 10.1016/j.isci.2022.105089

**Published:** 2022-09-07

**Authors:** Nadira Vadaq, Lisa van de Wijer, Louise E. van Eekeren, Hans Koenen, Quirijn de Mast, Leo A.B. Joosten, Mihai G. Netea, Vasiliki Matzaraki, André J.A.M. van der Ven

**Affiliations:** 1 Department of Internal Medicine, Radboudumc Center for Infectious Diseases, Radboud Institute of Health Science (RIHS), Radboud University Medical Center, Nijmegen, the Netherlands; 2 Center for Tropical and Infectious Diseases (CENTRID), Faculty of Medicine, Diponegoro University, Dr. Kariadi Hospital, Semarang, Indonesia; 3 Department of Laboratory Medicine, Laboratory of Medical Immunology, Radboud University Medical Center, Nijmegen, the Netherlands; 4 Department of Medical Genetics, Iuliu Haţieganu University of Medicine and Pharmacy, Cluj-Napoca, Romania; 5 Department of Immunology and Metabolism, Life and Medical Sciences Institute, University of Bonn, Germany

**Keywords:** Virology, Omics

## Abstract

Despite antiretroviral therapy (ART), people living with HIV (PLHIV) display persistent inflammation leading to non-AIDS-related co-morbidities. To better understand underlying mechanisms, we compared targeted plasma inflammatory protein concentration (n = 92) between a cohort of 192 virally suppressed PLHIV, who were followed-up for five years, and 416 healthy controls (HC). Findings were validated in an independent cohort of 649 virally suppressed PLHIV and 98 HC. Compared to HC, PLHIV exhibited distinctively upregulated inflammatory proteins, including mucosal defense chemokines, CCR5 and CXCR3 ligands, and growth factors. Unsupervised clustering of inflammatory proteins clearly differentiated PLHIV with low (n = 123) and high inflammation (n = 65), the latter having a 3.4 relative risk (95% confidence interval 1.2–9.8) to develop malignancies and trend for cardiovascular events during a 5-year follow-up. The best protein predictors discriminating the two inflammatory endotypes were PD-L1, VEGFA, LAP TGF β-1, and TNFRSF9. Our data provide insights into co-morbidities associated inflammatory changes in PLHIV on long-term ART.

## Highlights

• Targeted proteomics reveal upregulation of inflammatory proteins in PLHIV
• Upregulated inflammatory proteins in PLHIV are involved in specific pathways
• Unsupervised clustering differentiated PLHIV with high and low inflammation
• PLHIV with high inflammation had increased risk of malignancy and trend for developing CVD

## Graphical abstract

**Figure:**
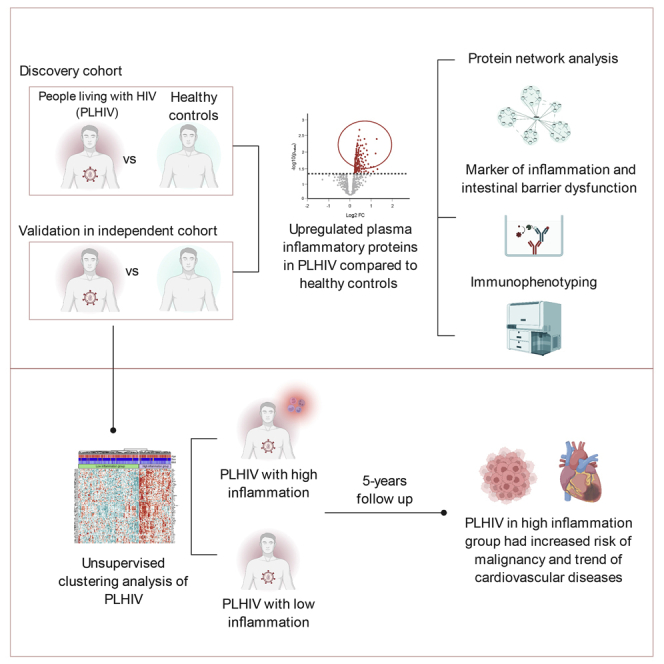

## Introduction

Combination antiretroviral therapy (cART) has dramatically increased the life expectancy of people living with HIV (PLHIV). Still, PLHIV have a higher risk of developing non-AIDS-related comorbidities, such as cardiovascular diseases (CVD) and malignancies than uninfected peers (Antiretroviral Therapy Cohort, 2017; Marcus et al., 2020). Persistent inflammation, possibly induced by low-level viremia, cART toxicity, co-infections, microbial dysbiosis, and translocation (Brenchley et al., 2006; Dinh et al., 2015; Gianella and Letendre, 2016; Llibre et al., 2012; van der Heijden et al., 2021), have been reported to contribute to the development of these long-term complications (Borges Á et al., 2013; Breen et al., 2011; Hunt et al., 2016). Increased concentrations of circulating inflammation markers, such as high-sensitivity C-reactive-protein (hsCRP), interleukin-6 (IL-6), tumor necrosis factor (TNF-α), and immune activation markers, including soluble (s)CD14 and sCD163 have been reported in virally suppressed PLHIV (Bastard et al., 2015; Hunt et al., 2016; Neuhaus et al., 2010; Novelli et al., 2020; van der Heijden et al., 2021).

Assessment of the profile of plasma inflammatory proteins allows the study of complex biological pathways that may lead to the identification of novel therapeutic targets and disease biomarkers. Previous studies that assessed plasma protein profiles were limited by small sample size and/or lack of a proper validation cohort (Babu et al., 2019a, 2019b; deFilippi et al., 2020; Lemma et al., 2020; Sperk et al., 2018; Vos et al., 2021). In addition, other studies included only patients with central obesity and insulin resistance using statins (deFilippi et al., 2020) or children with HIV infection (Lemma et al., 2020).

In the present study, we identified signatures of 92 inflammation-related plasma proteins in virally suppressed PLHIV (n = 841) and compared them to healthy controls (HC) (n = 514). Using a discovery cohort and an independent validation cohort, we found mostly upregulation of plasma inflammatory protein concentrations in PLHIV compared to HC. Furthermore, stratification of PLHIV based on the inflammatory proteome revealed two distinct clusters, one with a high- and the other with a low-inflammation profile.

## Results

### Characteristics of the study population

The discovery cohort consisted of 192 PLHIV and 416 HC that passed the quality control (QC) procedures (Table 1). Most (178/192 [93%]) of PLHIV were males, with a median (IQR) age of 52.4(13.3) years. PLHIV had median (IQR) CD4^+^ counts of 660 (330) cells/mL and were on ART for a median (IQR) 6.6 (7.2) years. Healthy controls were more often female (214/416 [51%], pvalue<0.0001), younger (median [IQR] age 23 [5] years, pvalue<0.0001), and leaner (median [IQR] BMI 22.3 [3.5] kg/m^2^, pvalue<0.0001) compared to PLHIV.

**Table 1:** General characteristics of the discovery cohort

Characteristic	PLHIV (n = 192)	HC (n = 416)	P-value
Age, years	52.4 (13.3)	23 (5.0)	<0.0001
Sex, female, n/N (%)	14/192 (7.3)	214/416 (51.4)	<0.0001
BMI, kg/m^2^	24.1 (3.9)	22.3 (3.5)	<0.0001
Time since HIV diagnosis, years	8.4 (8.5)	–	–
Time on ART, years	6.6 (7.2)	–	–
Nadir CD4^+^ cell count, cells/μl	250 (212.5)	–	–
Latest CD4^+^ count, cells/μl	660 (330)	–	–
Zenith HIV-RNA, copies/mL	100,000 (335,591)	–	–
Latest HIV-RNA, copies/mL	0 (40)		
Ratio CD4/CD8	0.7 (0.5)	–	–
HIV RNA blips 1 year^a^	5/192 (2.6)	–	–
HIV RNA blips 5 years^a^	33/192 (17.3)	–	–
ART classes, n/N (%)			
NNRTI	57/192 (29.7)	–	–
PI	28/192 (14.6)	–	–
INSTI	128/192 (66.7)	–	–
Co-medication, n/N (%)			
Cholesterol lowering drugs	51/192 (26.6)	–	–
Antihypertensive drugs	45/192 (23.4)	–	–
Antidiabetic drugs	9/192 (4.7)	–	–
Anti-inflammatory drugs	26/192 (13.5)	–	–
Anticoagulant	24/192 (12.5)	–	–
Vitamin D	44/192 (22.9)	–	–
Psychopharmaca	23/192 (12.0)	–	–
Co-morbidities, n/N (%)			
Cardiovascular disease	18/192 (9.4)	–	–
Hypertension	52/192 (27.2)		
Endocrine and metabolic disease	70/192 (36.5)	–	–
Respiratory disease	27/192 (14.1)	–	–
Gastrointestinal disease	23/192 (12.0)	–	–
Psychiatric conditions	47/192 (24.5)	–	–
Previously diagnosed malignancies	30/192 (15.7)	–	–
Fracture and bone disease	34/192 (17.8)	–	–
Lipodystrophy	30/192 (15.7)	–	–
Co-morbidities (5-year follow-up), n/N (%)			
Cardiovascular disease	18/192 (9.4)	–	–
Hypertension	25/192 (13.1)	–	–
Malignancies	14/192 (7.3)	–	–
Fracture and bone disease	29/192 (15.2)	–	–
Active smoking, n/N (%)	55/192 (28.6)	57/416 (13.7)	<0.0001

Data are depicted as median (IQR) unless stated otherwise. Data were analyzed using Mann-Whitney U or χ^2^ (or Fisher’s exact) where applicable.

PLHIV, people with HIV; HC, healthy controls; BMI, body mass index; ART, antiretroviral therapy; INSTI, integrase inhibitor; NNRTI, non-nucleoside reverse transcriptase inhibitor; PI, protease inhibitor.

a Viral blips defined as HIV-RNA >50 and <200 copies/mL preceded and followed by HIV-RNA ≤50 copies/mL in the 1 or 5 years before visit.

In the validation cohort, samples of 649 PLHIV and 98 HC passed the QC procedures (Table 2). PLHIV were slightly older than controls (median [IQR] age of 53 [15] years in PLHIV versus 49.5 [20.7] years in HC, pvalue = 0.001), and, although both groups predominantly consisted of males, the number of males was higher in the PLHIV group compared to the HC group (592/649 [91%] of PLHIV versus 74/98 [76%] of HC, pvalue<0.0001). All PLHIV in the validation cohort were on stable ART for a median (IQR) of 10 (9) years, with median (IQR) CD4^+^ counts of 700 (400) cells/uL.

**Table 2:** General characteristics of the validation cohort

Characteristic	PLHIV (n = 649)	HC (n = 98)	P-value
Age, years	53 (15)	49.5 (20.8)	0.001
Sex, female, n/N (%)	57/649 (8.7)	24/98 (24.5)	<0.0001
BMI, kg/m^2^	24.9 (4.8)	24.85 (3.6)	0.343
Time since HIV diagnosis, years	12 (12)	–	–
Time on ART, years	10 (9)	–	–
Latest CD4^+^ count, cells/μl,	700 (400)	–	–
Latest HIV-RNA, copies/mL	0 (2)	–	–

Data are depicted as median (IQR) unless stated otherwise. Data were analyzed using Mann-Whitney U or χ^2^ (or Fisher’s exact test) where applicable.

PLHIV, people with HIV; HC, healthy controls; BMI, body mass index; ART, antiretroviral therapy.

### Age, sex, and BMI influence plasma inflammatory protein concentrations in PLHIV and HC

We first explore the relationship among plasma inflammatory proteins in PLHIV and HC from the discovery cohort and found general strong positive correlations among plasma inflammatory proteins (Figure S3). The strongest associations were found among intracellular proteins (4E-BP1, STAMBP, AXIN1, ST1A1, SIRT2, and CASP8), chemokines associated with neutrophil (CXCL1, CXCL5, and CXCL6) and monocytes chemotaxis (MCP-2 and MCP4), natural killer cell surface receptor (CD244), and immune mediators related with T and B cells development and activation (IL7, CXCL11, CD40/TNRSF5 and TNFSF14).

Next, we examined the influence of host factors on plasma inflammatory protein concentrations in PLHIV and HC from the discovery cohort. Advancing age was associated with an overall higher concentration of inflammatory proteins in both groups (Figure S4). Female sex was significantly associated with reduced inflammatory proteins in HC, but not in PLHIV. The latter may have resulted from insufficient statistical power because of the small number of females in PLHIV (9%). BMI was positively associated with increased plasma inflammatory proteins concentrations, but to a lesser extent than age (Figure S4).

### Increased plasma inflammatory protein concentrations in PLHIV

**Figure 1:**
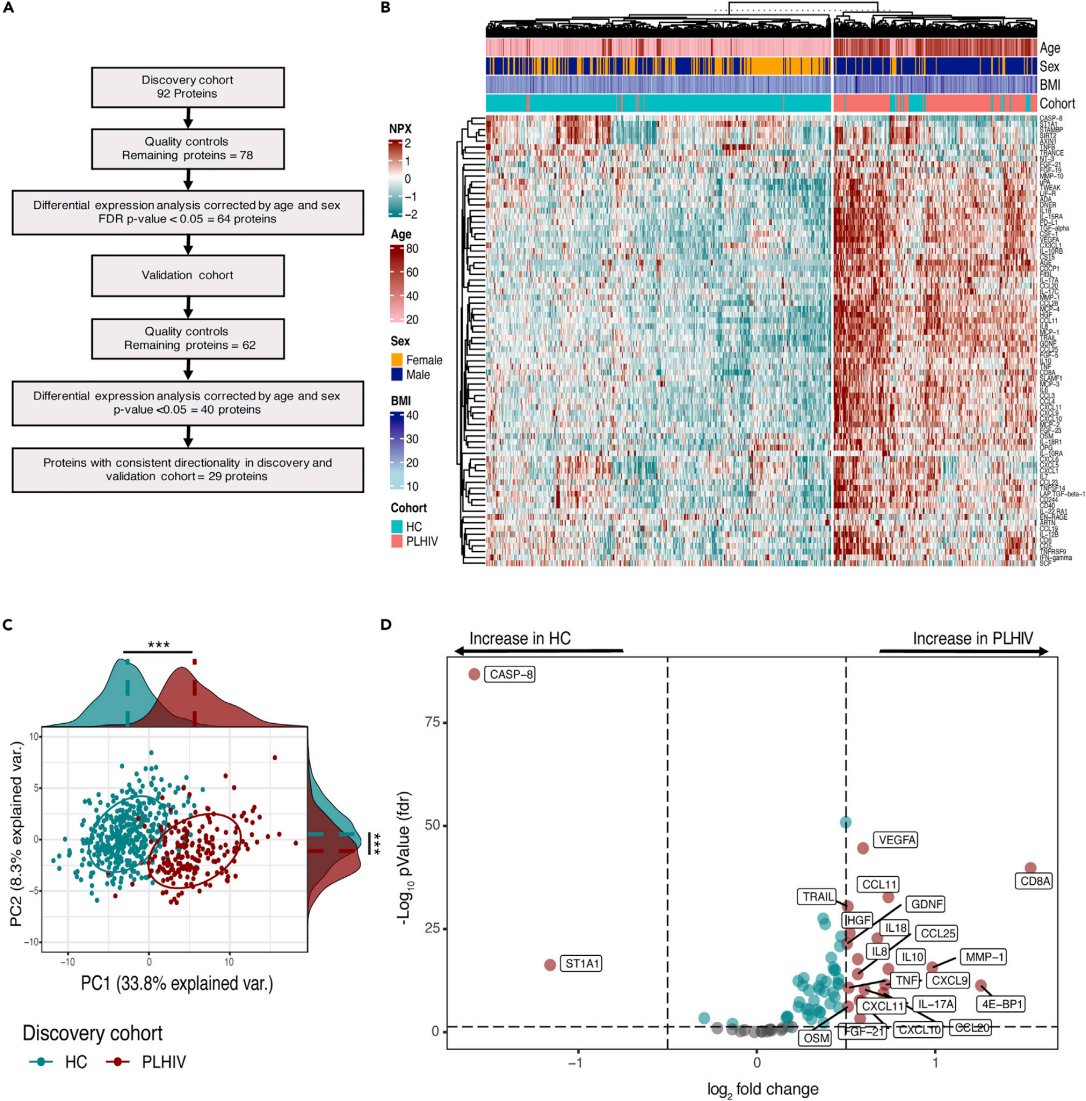
Differences of plasma inflammatory protein concentration between PLHIV and HC (A) Analytical process of DE plasma inflammatory protein analysis using a discovery and validation cohort, each consisting of PLHIV and HC. See also Figures S1 and S2. (B) Unsupervised k-means clustering of individuals from the discovery cohort of PLHIV (n = 188) and HC (n = 396) with complete demographic information using the relative concentration of plasma inflammatory proteins (n = 78). Data are shown as scaled log2 NPX values. The color code indicates the relative concentration of proteins across the samples of the two groups. Red and green colors indicate high and low protein concentrations respectively. Age, sex, BMI, and cohort group are presented on a color-coded scale. (C) PCA of plasma inflammatory proteins (n = 78) from the discovery cohort of PLHIV (n = 192) and HC (n = 411) using the first two principal components. The ellipses were centered based on the median of PC1 and PC2 for each group (PLHIV and HC). Protein distributions across PC1 and PC2 for each group are presented in marginal histogram plots. The median differences of the protein distribution across PC1 or PC2 between PLHIV and HC were calculated by the Mann-Whitney-U test. ∗∗∗p-value<0.0001. (D) Volcano plot of DE of proteins (n = 78) between PLHIV (n = 192) and HC (n = 404) from the discovery cohort. The analysis was performed using a linear regression model with age and sex as covariates. Fold change in the xaxis refers to the difference in the mean of log2 NPX values between PLHIV and HC. Only proteins that show FDR<0.05 (-log10 pvalue > 1.3) and log2 fold change >0.5 were annotated. Log2 fold change value of 1 means twice as high of relative protein concentration. See also Figure S5.

To compare the inflammatory profile between virally suppressed PLHIV and HC, we first performed differential expression (DE) analysis using 78 circulating inflammatory proteins measurements from the discovery cohort of PLHIV (n = 192) and HC (n = 416). Subsequently, we validated the significant results from the discovery cohort using a second independent cohort of PLHIV (n = 649) and HC (n = 98). The analytical process of DE plasma inflammatory protein analysis is depicted in Figure 1A.

First, we performed an unsupervised hierarchical clustering analysis using 78 circulating inflammatory proteins measurements from the discovery cohort (Figure 1B). We observed a distinct separation between the majority of PLHIV and HC individuals, suggesting an overall difference in their inflammatory profiles. This observation was confirmed through PCA showing separate clusters between PLHIV and HC (Figure 1C). We next performed DE analysis to assess differences in individual plasma inflammatory protein concentrations between PLHIV and HC from the discovery cohort. Given the effect of age and sex on the inflammatory protein concentrations (Figure S4), DE analysis was performed using a linear model with age and sex as covariates. The results of the DE analysis are presented in a volcano plot (Figure 1D). In total, 64 out of 78 proteins concentrations (82%) were differentially expressed (FDR<0.05) between PLHIV and HC, and most of the statistically significant proteins were upregulated in PLHIV. We found similar results when DE analysis between PLHIV and HC was performed with age, sex, BMI, and smoking status as covariates (Figure S5).

**Figure 2:**
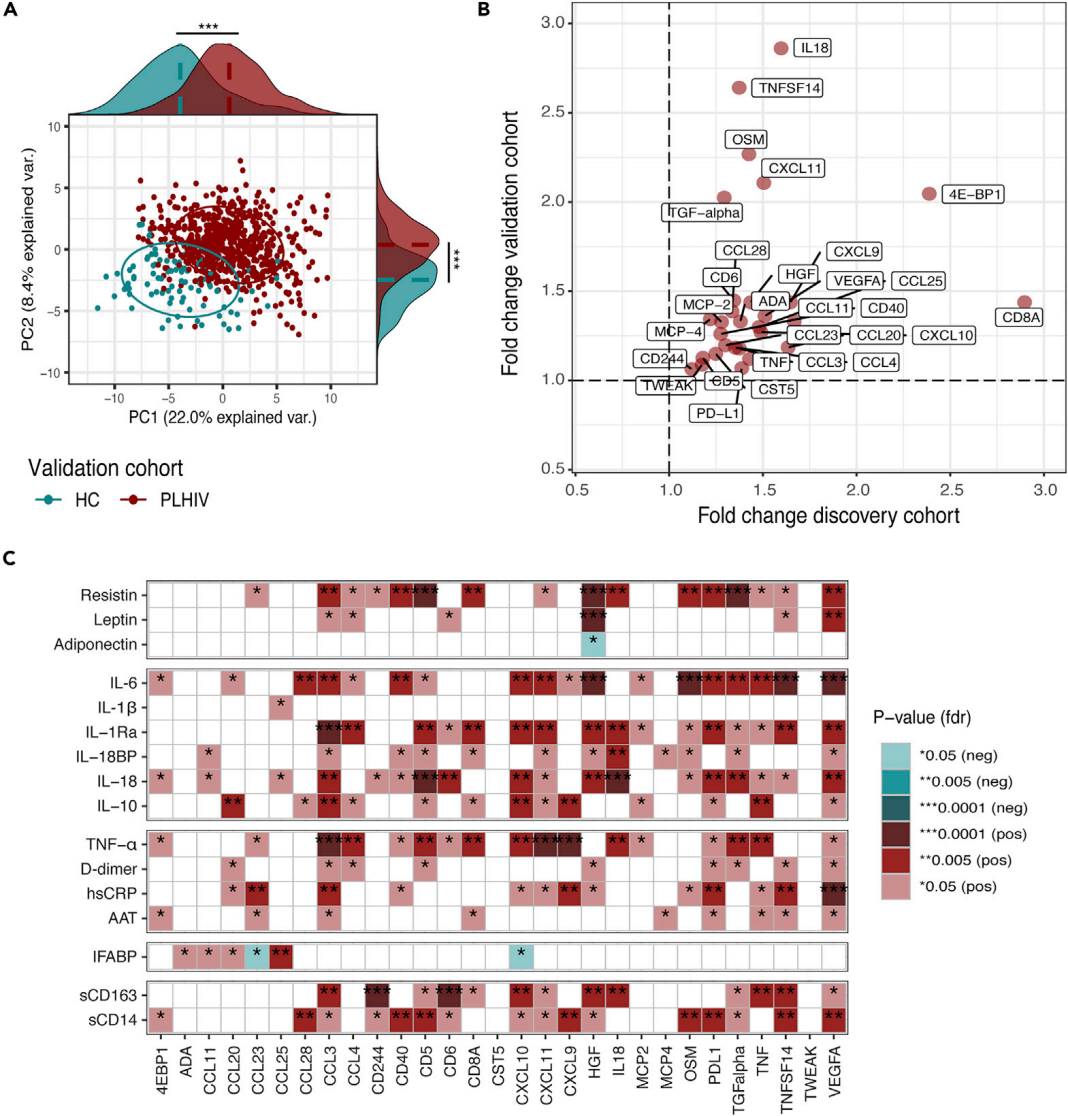
Validation of differentially expressed proteins between PLHIV and HC (A) PCA of plasma inflammatory proteins (n = 64) from the validation cohort of PLHIV (n = 98) and HC (n = 649) using the first two principal components. The ellipses were centered based on the median of the PC1 and PC2 for each group (PLHIV and HC). Protein distributions across PC1 and PC2 for each group are presented in marginal histogram plots. The median differences of the protein distribution across PC1 or PC2 between PLHIV and HC were calculated by the Mann-Whitney-U test. ∗∗∗p-value<0.0001. See also Figure S6. (B) Four-quadrant plot of the fold change of DEP (n = 29) in the discovery (xaxis) and validation cohort (yaxis). DE analysis was performed using a linear regression model with age and sex as covariates. See also Table S1 and Figures S6 and S7. (C) Heatmaps showing the correlations between the relative concentration of DEP (n = 29) and absolute concentration of plasma inflammatory markers measured in PLHIV of the discovery cohort. The analysis was performed by linear regression model using age and sex as covariates.

We confirmed our findings in the independent validation cohort of 649 PLHIV and 98 HC (Figure S1). In the validation cohort, PCA using relative concentrations of 62 proteins showed a different inflammatory profile between PLHIV and HC (Figure 2A). DE analysis in the validation cohort identified 40/62 (64.5%) DEP between PLHIV and HC with pvalue<0.05 (Figure S6), of which 29/40 (72.5%) proteins were upregulated in both the discovery and validation cohort (Figure 2B and Table S1).

Finally, we analyzed whether the relative concentration of the validated differentially abundant plasma inflammatory proteins (n = 29) measured by Olink was associated with the absolute concentration of plasma markers measured by ELISA. In general, DEP were positively associated with acute-phase proteins (TNF-α and hsCRP), adipokines (resistin), cytokines (IL-6, IL-1Ra, IL-18BP, IL-18, and IL-10), and monocyte activation markers (sCD14 and sCD163) (FDR<0.05) (Figure 2C). As expected, relative concentrations of IL-18 and TNF-α from the DE analysis significantly correlated with the absolute concentrations of IL-18 and TNF-α (FDR<0.005). In addition, hepatocyte growth factor (HGF) showed a significant positive correlation with other adipokines, such as resistin and leptin, and a negative correlation with adiponectin (Figure 2C).

### Network analysis reveals upregulation of specific inflammatory pathways in PLHIV

**Figure 3:**
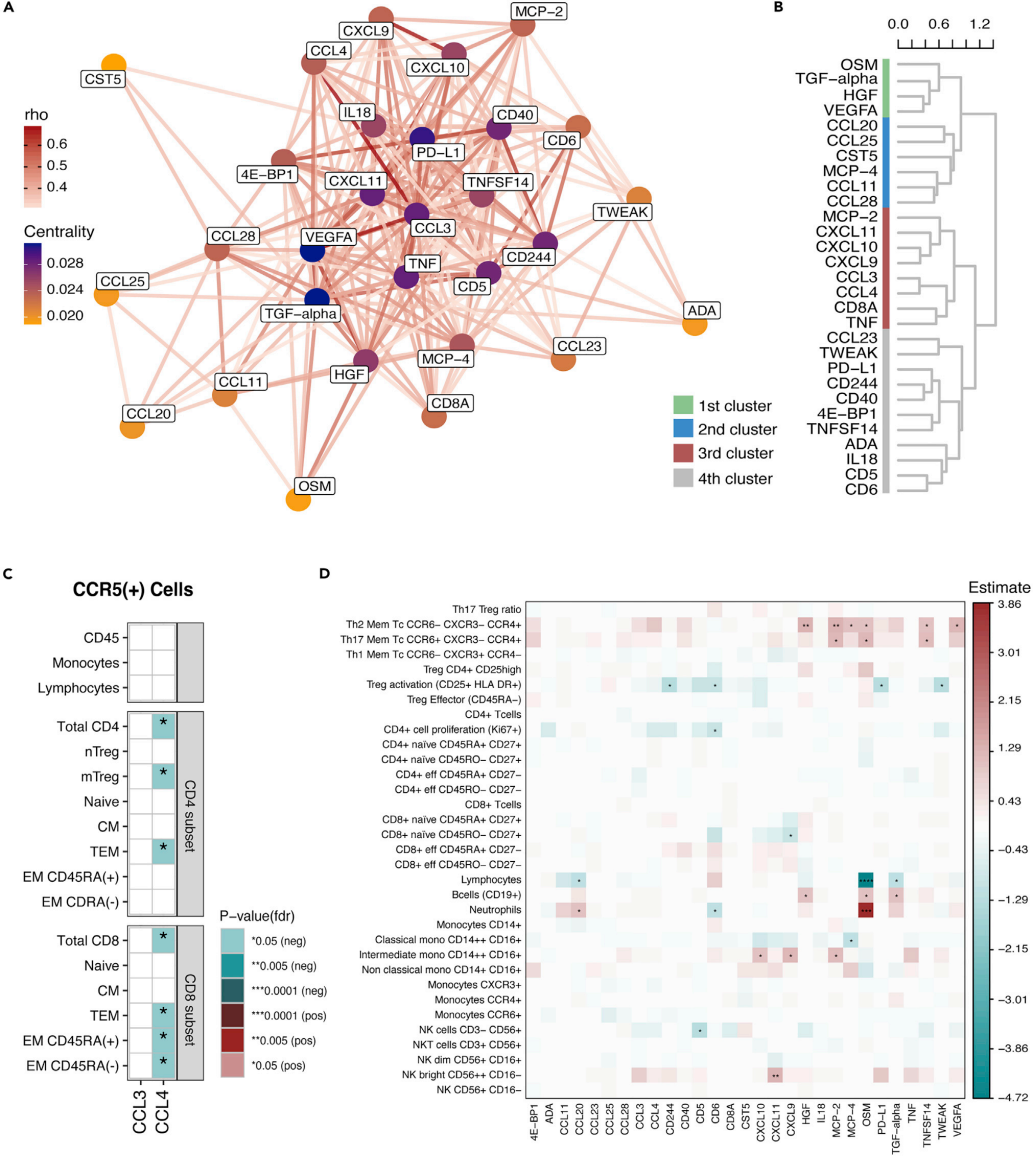
Dysregulation of distinct inflammatory pathways in PLHIV (A) Results from the network analysis using the DEP (n = 29) between PLHIV and HC. Network analysis was performed by calculating the spearman’s rank correlation between pairs of proteins measured in PLHIV of the discovery cohort (n = 192), and only those pairs with rho≥0.3 are presented. Proteins are represented as nodes, line colors connecting the nodes represent the degree of correlation for protein linkage; the darker the color, the stronger the correlation. Nodes color represents the centrality (importance) of protein based on nodes closeness. (B) Dendrogram of the DEP (n = 29) between PLHIV and HC. (C) Heatmap showing the correlations between the two CCR5 ligands (CCL3 and CCL4) and CCR5 expression measured in a different subset of immune cells in PLHIV of the discovery cohort. The correlations were calculated using a linear regression model with age and sex as covariates. (D) Heatmap showing the correlations between DEP (n = 29) and immunophenotyping data measured in PLHIV of the discovery cohort. The color-coding key depicts the beta estimate calculated by a linear regression model with age and sex as covariates. Significance level (FDR corrected) was defined as follows: <0.05(∗), <0.005(∗∗), and <0.0001(∗∗∗).

We further investigated the inter-relationship among the 29 significantly upregulated proteins in PLHIV compared to HC identified in the discovery and validation cohort. For this, we performed network analysis using relative concentrations of the 29 proteins from the PLHIV of the discovery cohort. Moderate to strong correlations (Spearman’s Rho>0.3) are displayed in Figure 3A. Overall, significant positive correlations were observed among DEP (FDR<0.05). Protein-protein interactions among DEP were further visualized by dendrogram based on hierarchical clustering analysis (Figure 3B). Four different clusters of proteins that shared similar functions were identified through the network and hierarchical clustering analysis (Figures 3A and 3B).

The first cluster consisted of the growth regulator oncostatin M (OSM) and several growth factors, including HGF, vascular endothelial growth factor A (VEGFA), transforming growth factor α (TGF-α). VEGFA and TGF-α were the most central proteins in the network analysis, indicating that these proteins showed the most pairwise correlations with other proteins (Figure 3A). OSM has been shown to stimulate the accumulation of immature and mature T-cells in lymph nodes, restoring immune responsiveness in immune-deficient mice (Clegg et al., 1996). Furthermore, OSM is known to play a role in the initiation and progression of Kaposi sarcoma (Miles et al., 1992; Nair et al., 1992), a common Herpes virus 8 related opportunistic cancer in PLHIV.

Furthermore, the second cluster consisted of the mucosal defense chemokines (chemokine (C–C motif) ligand 11 (CCL11), monocyte chemotactic protein 4 (MCP-4/CCL13), CCL20, CCL25, and CCL28)) and cystatin-D (CST5). Of interest, the concentrations of the three mucosal defense chemokines, CCL11, CCL20, and CCL25, were significantly associated with the absolute concentrations of IFABP, a marker of gut wall integrity (Figure 2C). In addition, CCL28 was associated with sCD14, a marker of monocyte activation (Figure 2C).

The third cluster consisted of CCR5 ligands (CCL3 and CCL4), C-X-C Motif Chemokine Receptor 3 (CXCR3) ligand chemokines (Chemokine (C-X-C motif) ligand 9 (CXCL9), CXCL10, CXCL11), MCP-2, cluster of differentiation 8A (CD8A), and TNF-α. CCR5 is known as the main HIV co-receptor, and we found a significant negative correlation between CCL4 concentrations with CCR5 expression of different CD4^+^ (total CD4^+^ cells, mTreg, and total pool effector memory cells) and CD8^+^ cell subsets (total CD8^+^ cells, total pool effector memory cells, and effector memory cells) (Figure 3C).

The last cluster consisted of an assortment of cytokine (IL-18), chemokine (CCL23), cluster of differentiation proteins (CD5, CD6, CD244, and PD-L1/CD274), eukaryotic translation initiation factor 4E-binding protein 1 (4E-BP1), and adenosin deaminase (ADA). Most of these proteins are known to play an important role in T cell activation, differentiation, and chemotaxis for T cell migration. Other members of this cluster were tumor necrosis factor superfamily (tumor necrosis factor superfamily member 14 (TNFSF14), and TWEAK/TNFSF12) and tumor necrosis factor receptor superfamily members (CD40). TNFSF14 is known as herpes virus entry mediator ligand (Montgomery et al., 1996), also for cytomegalovirus, a common co-pathogen in PLHIV.

To identify the cellular origin of our differentially expressed proteins (n = 29), we used single-cell transcriptomic publicly available data from the Human Proteomic Atlas (HPA) project (Karlsson et al., 2021). HPA used consensus transcriptomics data in 76 single cell types to classify genes according to their single-cell type-specific category. We found innate (dendritic cells, macrophages, langerhans cells, natural killer (NK) cells) and adaptive immune cells (T cells) among cells that produce most of our differentially expressed proteins (Figure S7).

Lastly, given that many of the identified proteins are primarily released by immune cells modulating subsequently their proliferation and migration, we investigated whether the DEP correlated with the proportion of circulating immune cells. The strongest association was found for OSM, which was positively and negatively associated with neutrophils and lymphocytes percentages respectively (Figure 3D). These findings are consistent with a previous study showing that OSM is primarily expressed in neutrophils and stored in neutrophils granules in the circulation (Uriarte et al., 2008). In addition, increased proportions of Th2, and Th17 cells were associated with higher HGF, MCP-2, MCP-4, OSM, TNFSF14, and VEGFA. A significant and positive correlation was also found between NK bright cells proportion with CXCL11, which is a chemotactic factor for activated T-cells.

Unsupervised clustering of plasma inflammatory protein concentrations revealed two distinct clusters of PLHIV with high and low inflammation profile.

**Figure 4:**
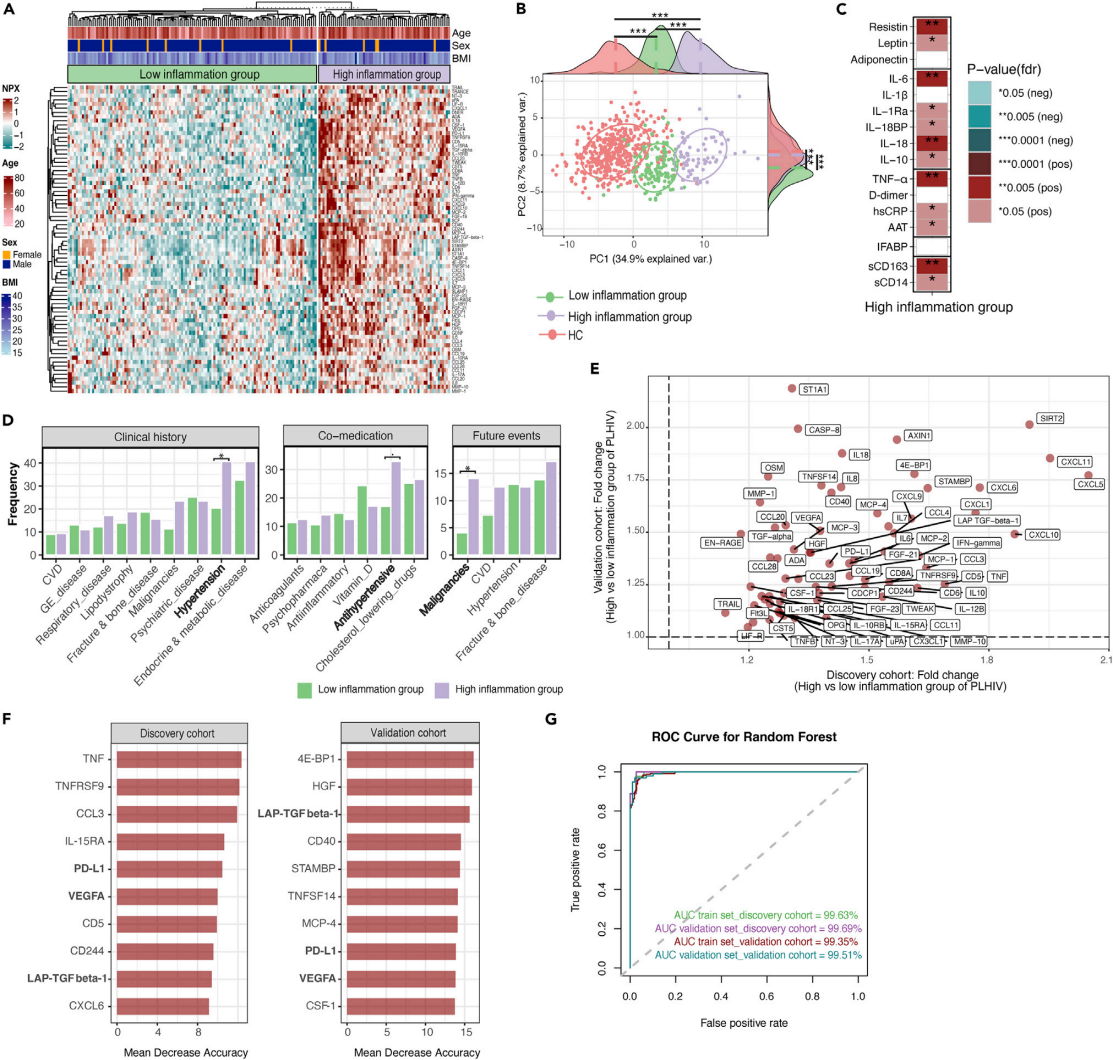
Clustering analysis of plasma inflammatory profiles and the relation with clinical events during 5-year follow-up in PLHIV (A) Unsupervised k-means clustering of PLHIV using the plasma inflammatory proteins (n = 74) measured in PLHIV of the discovery cohort (n = 188). Data are shown as scaled log_2_ NPX values. The color code indicates the relative concentration of proteins across the samples of the two clusters. Red and green colors indicate high and low protein concentrations, respectively. Age, sex, BMI are presented on a color-coded scale. See also Figure S9. (B) PCA of plasma inflammatory proteins (n = 74) measured in the discovery cohort using the first two principal components. Each dot represent participant from the HC (n = 415), and two clusters of PLHIV (low (n = 123) and high inflammation group (n = 65)). The ellipses were centered based on the median of the PC1 and PC2 for each group (HC, low, and high inflammation group). Protein distributions across PC1 and PC2 for each group are presented in marginal histogram plots. The median differences of the protein distribution across PC1 or PC2 between PLHIV and HC were calculated by Mann-Whitney-U test. ∗∗∗p-value<0.0001. (C) Heatmap presenting FDR corrected pvalues of the comparison between the absolute concentration of plasma inflammatory markers in high inflammation group versus low inflammation group. The analysis was performed by linear regression model using age and sex as covariates. (D) Barplot showing the frequency (yaxis) of comorbidities and co-medication at the baseline, and a 5-year follow-up clinical events in two clusters of PLHIV of the discovery cohort. The analysis was performed using the binomial logistic regression model using age and sex as covariates. ∗p-value<0.05. See also Figure S8 and Table S2. (E) Four-quadrant plot showing the fold change of DEP (n = 67) between high versus low inflammation group in PLHIV of the discovery (xaxis) and validation cohort (yaxis). Comparison of relative concentration of proteins between high and low inflammation group was performed using a linear regression model with age and sex as covariates. See also Table S3 and Figure S9. (F) Mean decrease accuracy of predictors for the high and low inflammatory endotypes in PLHIV of the discovery and validation cohort. Prediction model was performed using random forests classification model using demographic factors (age, sex, and BMI) and concentration of DEP between the high and low inflammation group (discovery [n = 71] or validation cohort [n = 69]) as predictors. Only the top 10 predictors based on their respective mean decrease accuracy values were visualized. (G) Receiver operating characteristic (ROC) analysis of the model’s performance in the train and validation sets of the discovery and validation cohort.

To assess the heterogeneity of plasma inflammatory proteins concentrations (n = 74) among PLHIV, we performed unsupervised hierarchical clustering analysis using the protein measurements (n = 74) of PLHIV from the discovery cohort. We identified two distinct clusters of PLHIV, one with low and one with a high inflammation profile (Figure 4A). Seventy-one out of 74 plasma inflammatory proteins were significantly upregulated in the high inflammation group (FDR<0.05) compared to the low inflammation group. In addition, PCA using the first two principal components showed a limited overlap between the two clusters of PLHIV and HC, with individuals from the low inflammation group showing an inflammatory profile between HC and the high inflammation group (Figure 4B). Furthermore, the absolute concentrations of plasma inflammatory markers were significantly higher in the high inflammation group compared to the low inflammation group, including acute phase proteins (TNF-α, hsCRP and AAT), adipokines (leptin and resistin), cytokines (IL-6, IL-1Ra, IL-18BP, IL-18, and IL-10), and monocyte activation markers (sCD14 and sCD163) (Figure 4C).

PLHIV of the high inflammation group were significantly older (median 55.4 years) compared to the low inflammation group (median 50.6 years) (pvalue<0.05). Of note, the distinct inflammatory clusters found within PLHIV was not explained by sex and BMI (Figure 4A), nor by HIV-related parameters, such as CD4 nadir and latest, CD4/CD8 ratio, HIV RNA zenith and latest value, viral blips, HIV and ART duration (Figure S8). Of importance, participants in the high inflammation group had more hypertension (pvalue<0.05) and a trend for previously diagnosed malignancies than those in the low inflammation group (Figure 4D). Compared to the low inflammation group, PLHIV in the high inflammation group used more anti-hypertension drugs (pvalue = 0.06) and less vitamin D at inclusion (Figure 4D). After inclusion, PLHIV from the discovery cohort were followed-up and relevant medical events were noted. After a 5-year follow-up period, we observed that PLHIV in high inflammation group developed more malignancies (13.8 versus 4%; relative risk (RR) 3.4; 95% confidence interval (CI) 1.2 to 9.8) and had a trend for more CVD events (12.3 versus 7.3%; RR 1.7; 95% CI 0.6 to 4.2) compared to PLHIV in low inflammation group (Figure 4D). Being in the high inflammation group was associated with an increased risk of malignancies even after controlling for age and sex (pvalue<0.05; binomial logistic regression model using age and sex as covariates) (Figure 4D). Details for the type of malignancy and CVD events seen during 5-year follow-up in high and low inflammation group of PLHIV were described in Table S2.

Clear separation of the PLHIV into a low and high inflammation group was also observed in the validation cohort, mirroring the heterogeneity of inflammatory protein concentrations in well-treated PLHIV (Figure S9). Sixty-nine proteins were upregulated in the high inflammation group compared to the low inflammation group from the validation cohort, of which 67 proteins overlapped with those found in the discovery cohort (Table S3 and Figure 4E). The range of fold change of these 67 DEP identified in the high versus low inflammation group of PLHIV was comparable to the range of fold change of 29 DEP identified in PLHIV versus HC (Figure 4E). Next, to identify the best protein predictors discriminating the high and low inflammation groups, we applied a random forest classification model in PLHIV from the discovery and validation cohort. The model was built using DEP and host demographic factors (age, sex, and BMI) as the input predictors. The predictors were ordered according to the mean decrease accuracy values, representing the importance of variables on distinguishing PLHIV of the high and low inflammation clusters. Out of the ten top-ranked predictors, three proteins were overlapped between the discovery and validation cohort (Figure 4F). These proteins include PD-L1, VEGFA, and latency-associated peptide transforming growth factor beta-1 (Lap TGF β-1). The classification model performance was tested by calculating the area under the curve (AUC) of the receiver operating characteristic (ROC) curve, yielding an AUC value of about 99% in train and test sets of the discovery and validation cohorts, respectively (Figure 4G).

### Association of plasma inflammatory proteins with HIV-related parameters and smoking

Many factors may influence inflammation, such as HIV-related clinical parameters, comorbidities, co-medication, and smoking. We, therefore, correlated the plasma inflammatory proteins (n = 74) from PLHIV of the discovery cohort with HIV-related parameters (CD4 nadir and latest, CD4/CD8 ratio, HIV RNA zenith and latest, viral blips, HIV and ART duration, and HIV medication), HIV reservoirs, comorbidities, co-medication, and history of smoking in PLHIV from the discovery cohort.

First of all, we found strong positive associations between fibroblast growth factor 23 (FGF-23) and IL-6 concentrations with HIV duration (pvalue<0.005) (Figure S10A). Moreover, apart from higher FGF-23, CCL19, and OSM (FDR<0.05), PLHIV with a history of CVD also had higher signaling lymphocytic activation molecule family member 1 (SLAMF1) and HGF (pvalue<0.05) compared to those that were not having a history of CVD. Circulating concentrations of CST5, an early biomarker for traumatic brain injury (Hill et al., 2017), were significantly increased in PLHIV with psychiatric conditions (pvalue<0.005) (Figure S10B). Regarding co-medication, participants using antihypertensive and anticoagulant drugs had increased concentrations of FGF-23, CCL19, OSM, and HGF (pvalue<0.05) (Figure S10C). Of importance, increased concentrations of several inflammatory proteins, including OSM and SLAMF1 (pvalue<0.005), as well as FGF-23, HGF, and CCL19 (pvalue<0.05), were significantly associated with a higher incidence of CVD during a 5-year follow-up period. In addition, tumor necrosis factor receptor superfamily member 9 (TNFRSF9) was significantly associated with the development of malignancies during a 5-year follow-up period (pvalue<0.005) (Figure S10D). Furthermore, we observed comparable plasma inflammatory profiles between different ART groups (INSTI, NNRTI, and PI), as shown by PCA using the protein measurements of 74 inflammatory proteins (Figure S10E). This observation is in agreement with a previous study in well-controlled PLHIV with dyslipidemia (deFilippi et al., 2020).

**Figure 5:**
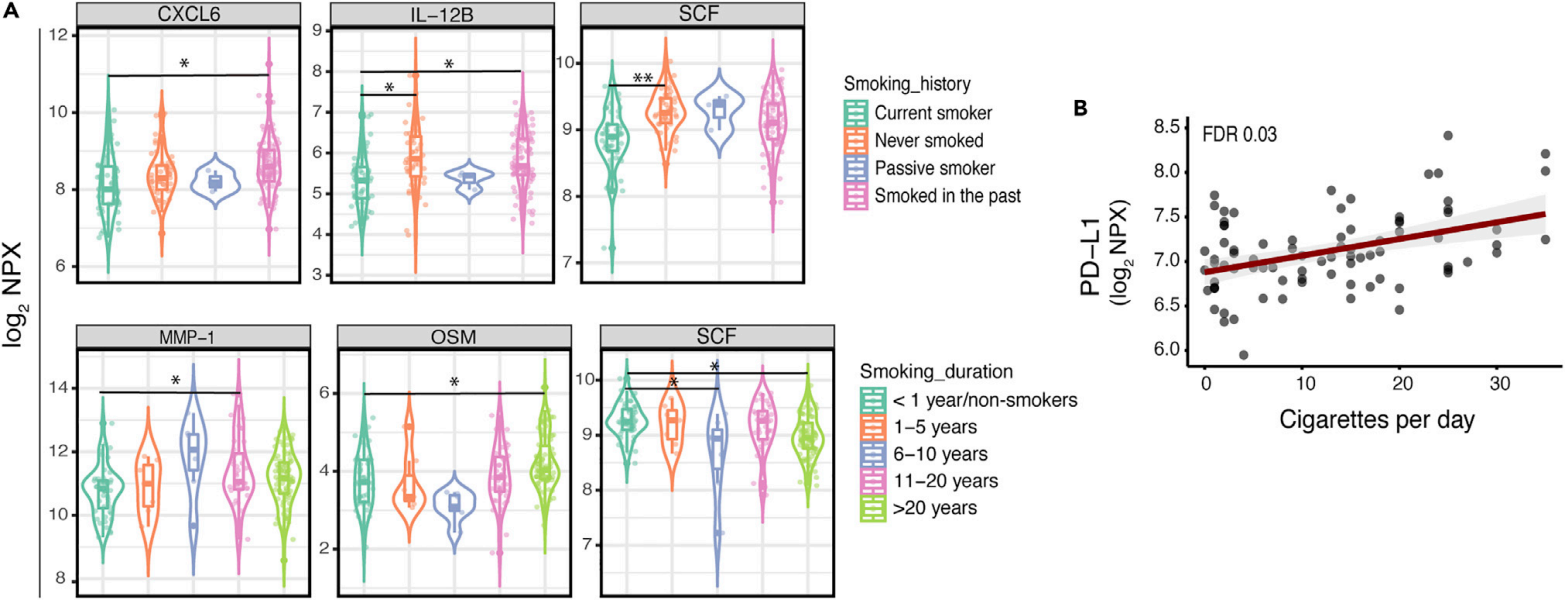
Association between plasma inflammatory proteins and smoking history in PLHIV (A) (Upper) Violin boxplot showing the comparison CXCL6, IL-12B, and SCF in different smoking history categories in PLHIV of the discovery cohort. (Lower) Violin boxplot showing the comparison MMP-1, OSM, and SCF in different smoking duration categories in PLHIV of the discovery cohort. The analysis was performed by linear regression model using age and sex as covariates. In all boxplots, the in-box line defines the median value, hinges depict 25^th^ and 75^th^ percentiles and whiskers extend to ±1.5 interquartile ranges; each dot indicates an individual participant. Significance level was set by FDR<0.05(∗), <0.005(∗∗), and <0.0001(∗∗∗). (B) Scatterplot showing an association between PDL-1 and number of cigarettes smoked per day in PLHIV; each dot indicates an individual participant.

Finally, we investigated the influence of smoking on plasma inflammatory proteins in PLHIV using our discovery cohort (Figures 5A and 5B). PLHIV were grouped based on smoking history (non-smokers, currently active smokers, passive smokers, and smoked in the past) and duration of smoking (those that do not smoke/with <1, 11–20, 6–10, and >20 years of active smoking). Non-smokers had a significantly higher stem cell factor (SCF) (FDR<0.005) and IL-12B (FDR<0.05) compared to a currently active smoker. CXCL6 and IL-12B concentrations were significantly higher in those who smoked in the past compared to the currently active smokers (FDR<0.05). In addition, matrix metallopeptidase 1 (MMP-1) and OSM concentrations were significantly increased in participants with 11–20 years of smoking or those who smoked for more than 20 years compared to those who never smoked or smoked for less than one year, respectively (FDR<0.05) (Figure 5A). Consistent with previous results, SCF concentrations were lower in participants with 6–10 years and more than 20 years history of smoking compared to those who never smoked or smoked for less than one year (FDR<0.05) (Figure 5A). PD-L1 was positively associated with the number of cigarettes smoked per day (FDR<0.05) (Figure 5B). Altogether, these findings corroborate the pro-inflammatory effect of cigarette smoking on the concentrations of a limited number of proteins (n = 6) in PLHIV. Given the limited influence of smoking on plasma inflammatory proteins, the DE analysis between PLHIV and HC showed comparable results before and after adjustment for smoking status in the discovery cohort (Figure S5).

## Discussion

We profiled 92 circulating inflammatory proteins in the plasma of 192 virally suppressed PLHIV and 416 HC of European ancestry and further validated our findings in a second independent cohort. This is the first study that comprehensively assessed the inflammatory profile in PLHIV using a broad-scale proteomic approach. Previous studies identified only a limited number of inflammatory biomarkers in PLHIV. For example, nine inflammation markers were found to be differentially expressed in a study of 185 well-treated PLHIV and 104 HC of South African ancestry, with only three proteins (CCL25, PD-L1, and CXCL10) being in agreement with our findings (FDR<0.05, same direction) (Vos et al., 2021). Furthermore, two studies by Babu et al. in 53 PLHIV and 41 HC and 22 PLHIV and matched HC reported changes in 11 (pvalue<0.01) and 3 (FDR <0.1) proteins in well-treated PLHIV compared to HC respectively. Only five (ADA, CD8A, 4E-BP1, CCL23, and CST5) and one (CD8A) protein were similar to our findings respectively (Babu et al., 2019a, 2019b). Another study using a cohort of PLHIV with dyslipidemia (n = 89) and HC (n = 46) also showed little overlap with our results, with only four inflammatory proteins (4E-BP1, ADA, TNFSF14, and CD40) overlapping with our results (FDR<0.05, same direction) (deFilippi et al., 2020). Of note, a study using 43 children with HIV infection and matched controls showed 15 proteins being downregulated in children with HIV infection compared to HC (FDR<0.01), with none of the proteins being identified in a similar direction compared to our study (Lemma et al., 2020). These differences between studies can be attributed to different cohort characteristics, especially the ethnicities, number of participants, proteins tested, and analysis strategies. Notably, one major disadvantage of the earlier studies is the small number of PLHIV and healthy controls studied, leading to a limited statistical power to identify true changes. In our study, we included a large number of subjects, confirmed the pro-inflammatory status of the PLHIV in the discovery cohort (van der Heijden et al., 2021), and found that the upregulated DEP in PLHIV strongly associated with the absolute concentration of plasma inflammatory markers, such as TNF-α, IL-6, and monocyte activation markers (sCD14 and sCD163), which have been previously described as predictors of CVD and mortality in PLHIV (McKibben et al., 2015; Sandler et al., 2011).

Most of our DEP were derived from innate immune cells (Figure S7), corroborating the functional role of circulating innate immune cells on the persistent inflammation in PLHIV on ART (Altfeld et al., 2011; Altfeld and Gale, 2015; Rustagi and Gale, 2014). We previously reported increased monocyte-derived cytokine responsiveness in the same cohort of PLHIV (van der Heijden et al., 2021). Among DEP identified in this study are mucosal defense chemokines (CCL11, MCP-4, CCL20, CCL25, and CCL28) which are upregulated in PLHIV. Changes in mucosal defense chemokines, which are pivotal to maintain intestinal barrier function (Kulkarni et al., 2017; Sokol and Luster, 2015) may be a consequence or a result of the disruption of the intestinal immune system. The association of the intestinal immune system and HIV infection has great interest, and the assessment of the intestinal mucosal immune system has provided novel directions for therapeutic interventions that modify the consequences of acute HIV infection (Brenchley and Douek, 2008). Also, chronic microbial translocation in well-treated PLHIV because of intestinal damage has been linked with persistent immune activation (Brenchley et al., 2006; Dinh et al., 2015; van der Heijden et al., 2021). Given the importance of the intestinal mucosal immune system in HIV infection, we found a strong association between an inflammatory marker for the intestinal lymphocytes recruitment, CCL25, and a marker for intestinal barrier function, that is, IFABP, indicating a role of the intestinal barrier in the inflammatory status of PLHIV.

Furthermore, CCL3 and CCL4 chemokines that were upregulated in PLHIV are known to be ligands for CCR5, the main HIV co-receptor. We observed that CCL4 was negatively correlated with CCR5 expression in several subsets of CD4^+^ and CD8^+^ cells, suggesting protective effects against cell-to-cell HIV transmission (Figure 3C). It has been shown that the dose-dependent administration of the recombinant CCL3 and CCL4 inhibit different strains of HIV-1, and HIV-2 as well as simian immunodeficiency virus (SIV) *in vitro* (Cocchi et al., 1995). Given that CCR5 is predominantly found in CXCR3 expressing cells, we assumed that the upregulation of CXCR3 ligands in PLHIV (CXCL9, CXCL10, and CXCL11) might facilitate the recruitment of T cells and enhance virus propagation or development on CVDs (Li and Ley, 2015). Of interest, CXCL9, CXCL10, and CXCL11 have been reported to predict the progression of HIV disease in the primary HIV infection (Yin et al., 2019). Moreover, we identified growth factors and regulator proteins (HGF, VEGFA, TGF-α, and OSM) to be upregulated in PLHIV compared to HC. Higher concentrations of these proteins seem to reflect alteration in the regulation of many important cellular processes in PLHIV.

To identify whether there are subgroups within the PLHIV based on their plasma inflammatory profile, we performed unsupervised clustering using the discovery (n_PLHIV_ = 188) and validation cohort (n_PLHIV_ = 649). PLHIV were clustered into two groups: those with a low and high inflammatory profile (Figure 4A). The high inflammation group showed upregulation of 67 out of 74 proteins and increased absolute concentrations of circulating inflammatory markers compared to the low inflammation group. The upregulation of almost all inflammatory proteins may reflect a broad dysregulation of immune responses in the high inflammation group as these proteins are involved in various inflammatory pathways. Upregulation of intracellular proteins (4E-BP1, STAMBP, AXIN1, ST1A1, SIRT2, and CASP8) in the high inflammation group may suggest a higher intracellular protein leakage and/or cell death in this group, given strong interrelations between intracellular proteins with proteins involved in innate and adaptive immune response (Figure S3). Furthermore, the range of fold change of DEP in the high versus low inflammation group of PLHIV (Figure 4E) was similar to the fold change in PLHIV versus HC (Figure 2B), suggesting a comparable level of dysregulation of inflammation between these groups.

Compared to the low inflammation group, PLHIV in the high inflammation group had more hypertension at baseline and a higher number of people developing various types of malignancies (Table S2) (RR 3.4; 95% CI 1.2 to 9.8) and a trend of CVD (RR 1.7; 95% CI 0.6 to 4.2) during a 5-year follow-up period (Figure 4D). This association appears to be independent of age, HIV-clinical parameters (CD4 nadir and latest, CD4/CD8 ratio, HIV RNA zenith and latest, viral blips, HIV and ART duration, and HIV medication), and other recognized pro-inflammatory risk factors (obesity and smoking status). Of note, although the high inflammation group were older than those in the low inflammation group, age has not been observed as one of the top ten predictors that separated the two inflammatory endotypes in both cohorts (discovery and validation), indicating that the differences in the inflammation levels may not be primarily attributed to the age differences. Three proteins (PD-L1, VEGFA, and Lap TGFβ-1) appeared to be the best predictors discriminating the two inflammatory endotypes in the discovery and validation cohort (Figure 4F). PD-1 and PD-L1 inhibitors are well-known checkpoint anti-cancer drugs (Chen et al., 2016). TGF-β and VEGFA can induce the expression of PD-L1 and its receptors (PD-1), respectively (Du et al., 2021; Song et al., 2014; Voron et al., 2015), promoting immunosuppression during malignant transformation. Anti-cancer immunotherapy targeting either one or a combination of PD-L1, TGFβ or VEGF has demonstrated a synergistic anti-tumor effect and is subjected to ongoing research (Ciardiello et al., 2020; Courau et al., 2016; Gulley et al., 2021; Hack et al., 2020). The safety and efficacy of anti-PD1 or VEGF inhibitor therapy have been reported in studies of HIV patients with different types of malignancies (Bari et al., 2019; Bender Ignacio et al., 2016; Lavolé et al., 2018; Ostios-Garcia et al., 2018). In addition, TNFRSF9 showed the strongest association with the development of malignancy during a 5-year follow-up period (Figure S10D) and was among the top predictors discriminating the two inflammatory endotypes in PLHIV (Figure 4F). TNFRSF9 is an immune costimulatory receptor expressed on activated T- and natural killer (NK) cells and has been proposed as a new target for cancer immunotherapy. Prior studies have shown that utomilumab and urelumab, which are TNFRSF9 agonistic antibodies, can deliver costimulatory signals, enhancing T-cell–mediated anti-tumor activity *in vitro* and *in vivo* (reviewed in (Chester et al., 2018)).

Furthermore, FGF-23, OSM, CCL19, SLAMF1, and HGF consistently showed positive associations with CVD events in PLHIV, both at baseline and during a 5-year follow-up (Figure S10), confirming the well-established link of sustained inflammation in PLHIV under suppressive ART with an increased CVD risk (Freiberg et al., 2013; Nordell et al., 2014). Previous studies have highlighted the relation of these proteins with the pathophysiology (Cai et al., 2014; Kubin et al., 2011) and the incidence of CVD (Bell et al., 2016, 2018; Bielinski et al., 2017; Ferreira et al., 2019; Gruson et al., 2017; Ikeda et al., 2021; Ix Joachim et al., 2012; Paul et al., 2021; Vázquez-Sánchez et al., 2021) in the general population and PLHIV (Atta et al., 2016; Domingo et al., 2015; García-Broncano et al., 2014). In addition, OSM receptor has been reported to be associated with the presence of coronary calcium, independent from traditional atherosclerotic CVD risk, in PLHIV on ART (Kolossváry et al., 2022). Collectively, these findings support previous reports that increased inflammation may follow or induce non-AIDS comorbidities, and further pinpoint PD-L1, VEGFA, LAP TGFβ-1, and TNFRSF9 as possible targets to decrease the risk for malignancies in well-treated PLHIV.

In conclusion, our study underscores the importance of targeting specific inflammatory pathways that are upregulated in virally suppressed PLHIV compared to HC. Although upregulation of mucosal defense chemokines that represent disruption of intestinal immunity is well known in HIV infection, other inflammatory pathways, such as CCR5 ligands, CXCR3 ligands, and growth factors proteins, were less known and can pave the way toward new therapeutic options. Last but not least, clinicians should be aware that PLHIV with a high inflammation endotype are at increased risk to develop malignancies and CVDs. Therefore, patient stratification based on inflammatory profile warrants further research in order to develop better therapeutic and preventive strategies against malignancies and CVDs in virally suppressed PLHIV.

### Limitations of the study

There were several limitations to our study. First, the findings are mainly correlative that hinders causation inference as correlation does not imply causation. Second, demographic differences between groups across cohorts may introduce bias in the results of our study. However, all analyses were performed with adjustment for age and sex to account for the demographic differences. Next, a low percentage of participants with comorbidities may limit the power to find statistically significant associations with plasma inflammatory markers in PLHIV after accounting for multiple testing burden. However, we identified significant associations between DEP and different comorbidities before multiple testing correction (pvalue< 0.05), which is in agreement with previous studies.

## STAR★Methods

### Key resources table

**Table:** 

REAGENT or RESOURCE	SOURCE	IDENTIFIER
**Antibodies**		

CD16-FITC, 3G8	Beckman Coulter	Cat#IM0841U
HLA-DR PE, Immu-357	Beckman Coulter	Cat#IM1639U; RRID: AB_2876782
CD14 ECD, UCHT1	Beckman Coulter	Cat#A07748
CD4 PE-Cy5.5, 13B8.2	Beckman Coulter	Cat#B16491
CD25 PC7, M-A251	BD	Cat#557741; RRID: AB_396847
CD56 APC, N901	Beckman Coulter	Cat#IM2474; RRID: AB_130791
CD8 APC-AF700, B9.11	Beckman Coulter	Cat#A66332; RRID: AB_2750854
CD19 APC-AF750, J3-119	Beckman Coulter	Cat#A94681; RRID: AB_2833030
CD3 PB, UCHT1	Beckman Coulter	Cat#A93687; RRID: AB_2728095
CD45 KO, J33	Beckman Coulter	Cat#A96416; RRID: AB_2888654
CD45RA FITC, ALB11	Beckman Coulter	Cat#A07786
CD3 PE, UCHT1	Beckman Coulter	Cat#A07747
CD45RO ECD, UCHL1	Beckman Coulter	Cat#IM2712U; RRID: AB_10639537
CD27 PE-Cy5.5, 1A4CD27	Beckman Coulter	Cat#B21444
CD127 APC-AF700, R34.34	Beckman Coulter	Cat#A71116; RRID: AB_2889979
CD8 APC-AF750, B9.11	Beckman Coulter	Cat#A94683
CD4 PB, 13B8.2	Beckman Coulter	Cat#A82789; RRID: AB_2892549
CD19 APC-AF750, J3-119	Beckman Coulter	Cat#A94681; RRID: AB_2833030
CD3 ECD, UCHT1	Beckman Coulter	Cat#A07748; RRID: AB_1575956
KI67 (ic) FITC, B56	BD	Cat#561165; RRID: AB_10611866
CD45RA ECD, 2H4LDH11LD89	Beckman Coulter	Cat#IM2711U; RRID: AB_10640553
CD196 PE, 11A9	BD	Cat#559562; RRID: AB_397273
CD8 ECD, SFCI21Thy	Beckman Coulter	Cat#737659; RRID: AB_2751015
CD183 PerCp5.5, G025H7	Biolegend	Cat#353714; RRID: AB_10962908
CD194 PC7,1G1	BD	Cat#557864; RRID: AB_396907
CD25 APC, 2A3	BD	Cat#340907; RRID: AB_2819021
CD4 AF700, RPA-T4	eBioscience	Cat#56-0049-42; RRID: AB_11219085
CD197-BV421, G043H7	Biolegend	Cat#353208; RRID: AB_11203894

**Critical commercial assays**		

Olink® Target 96	Olink Proteomics	Olink® Target 96 Inflammation Panels
Olink® Explore panels	Olink Proteomics	Olink® Explore 1536
Human alpha-1 anti trypsin/Serpin A1 DuoSet ELISA kit	R&D Systems	Cat#DY1268
Human Adiponectin/Acrp30 DuoSet ELISA kit	R&D Systems	Cat#DY1065
Human C-Reactive Protein/CRP Quantikine ELISA Kit	R&D Systems	Cat#DCRP00
Human Total IL-18 DuoSet ELISA kit	R&D Systems	Cat#DY318
Human IL-18 BPa DuoSet ELISA kit	R&D Systems	Cat#DY119
Human Leptin DuoSet ELISA kit	R&D Systems	Cat#DY398
Human Resistin DuoSet ELISA kit	R&D Systems	Cat#DY1359
Human FABP2/I-FABP DuoSet ELISA kit	R&D Systems	Cat#DY3078
Human CD163 Quantikine ELISA kit	R&D Systems	Cat#DC1630
Human CD14 Quantikine ELISA kit	R&D Systems	Cat#DC140
IL-6, IL-1β, TNF-α, IL-10, and IL-1Ra Simple Plex cartridges using the Ella apparatus	Protein Simple	
Human D-Dimer ELISA kit	Abcam	Cat#ab260076

**Deposited data**		

RNA single cell type data	The Human Protein Atlas	http://www.proteinatlas.org/; RRID:SCR_006710

**Software and algorithms**		

R version 4.0.2	R Core Team	https://cran.r-project.org

**Other**		

Proteomics (OLINK) data from the Human Functional Genomics Project (HFGP)	HFGP	www.humanfunctionalgenomics.org
The 2000HIV project cohort data	The 2000HIV project	https://clinicaltrials.gov/ct2/show/NCT03994835)

### Resource availability

#### Lead contact

Further information and requests for resources and reagents should be directed to and will be fulfilled by the lead contact, Nadira Vadaq (N.Nadira@radboudumc.nl).

#### Materials availability

This study did not generate new unique reagents.

### Experimental model and subject details

#### Cohort of study participants

This study included discovery and an independent validation cohort of virally suppressed PLHIV and HC and is part of the Human Functional Genomics Project (HFGP) (www.humanfunctionalgenomics.org) (Netea et al., 2016). A schematic representation of the cohorts is shown in Figure S1.

#### Discovery cohort

The discovery cohort consisted of PLHIV recruited between December 2015 and February 2017 at Radboudumc, Nijmegen, the Netherlands. Participants were 18 years and older, received ART for more than six months, and had HIV-RNA levels <200 copies/ml. Detailed patient characteristics have been reported elsewhere (Van deWijer et al., 2021; van der Heijden et al., 2021). Relevant comorbidities and co-medication data with a prevalence of about 10% of total study participants were presented. PLHIV were followed for the development of clinical events during a period of five years (2016–2021). The control group consisted of a historical cohort of healthy Dutch individuals (500 Functional Genomic [500FG] cohort) recruited between August 2013 and December 2014 at Radboudumc, the Netherlands. Details about the 500FG cohort have been previously described (Li et al., 2016; ter Horst et al., 2016). In total, protein measurements were available for 198/211 of the PLHIV and 423/534 of the healthy controls.

#### Validation cohort

The HIV validation cohort consisted of the first consecutive 661 participants of the 2000HIV study (ClinicalTrials.gov: NCT03994835) enrolled between February 2019 and October 2021 in four different Dutch HIV treatment centers in Nijmegen, Amsterdam, Tilburg, and Rotterdam. Similar inclusion and exclusion criteria were applied for the discovery cohort. Healthy control samples were obtained from the 200 Functional Genomic (200FG) cohort, as described elsewhere (Li et al., 2016). Samples were collected between 2019 and 2020. In total, protein measurements were available for 661 of the PLHIV and 100 sex- and age-matched healthy controls.

#### Study approval

The studies involving human participants were reviewed and approved by the Ethical Committee of the Radboud University Medical Center Nijmegen, the Netherlands (NL42561.091.12 (200HIV and 500FG), NL68056.091.81 (2000HIV), and 2018-399 EC (200FG)). The patients/participants provided their written informed consent to participate in this study.

### Method details

#### Sample processing

Sample collection and processing in the cohorts of the HFGP are performed using similar study procedures (Netea et al., 2016). Blood samples of the discovery cohort were collected at Radboudumc and were processed immediately. For the validation cohort, blood samples from different centers were sent overnight to Radboudumc or left on the bench at room temperature to be processed the next day. The second and first freeze-thaw cycles of plasma fractions were used for proteomic analysis in the discovery and validation cohort, respectively. Samples were centrifuged using similar settings and stored at −80°C.

#### Proteomic profiling of circulating inflammatory proteins

For the discovery cohort, 92 unique protein biomarkers were measured using the Olink inflammation panel (Olink Proteomics, Uppsala, Sweden) (Assarsson et al., 2014). For the validation cohort, the Olink Explore panel (Filbin et al., 2021) (n = 92/1472 proteins) was used. PLHIV and healthy controls were measured simultaneously. Proximity extension assay (PEA) technology was applied to measure relative concentration of the proteins, which were presented as log_2_ normalized protein expression level (NPX) values.

#### Plasma inflammatory markers

In all PLHIV from the discovery cohort, absolute concentration of interleukins (*IL-18* and *IL-18BP*), acute phase proteins (hsCRP, D-dimer, and α-1 anti-trypsin (AAT)), adipokines (leptin, adiponectin, and resistin), intestinal barrier dysfunction marker (intestinal fatty acid-binding protein (IFABP)), and monocyte activation markers (sCD14 and sCD163) were measured using ELISA (Duoset or Quantikine, R&D Systems; Abcam, Cambridge, MA, USA) according to the manufacturer’s protocols. Concentrations of IL-6, IL-1β, TNF-α, IL-10, and IL-1Ra were measured using SimplePlex Cartridges (Protein Simple).

#### Immunophenotyping and gating strategies

Immunophenotyping data were available for PLHIV from the discovery cohort. The complete protocol of immunophenotyping and gating strategies has been described before (Van deWijer et al., 2021). Samples were measured on a 10-color Navios flow cytometer (Beckman Coulter, Fullerton, CA, USA) equipped with 488, 638, and 405 nm solid-state lasers. Stained blood samples were analyzed using five supplemental 10-color antibody panels. Flow cytometry data were analyzed using Kaluza software version 1.3 and version 2.1. Gating was conducted and verified by two independent specialists. The absolute number of white blood cells (WBC) per mL of blood (by Beckman Coulter AcT Diff Hematology Analyzer) were used to calculate absolute numbers of leukocyte (CD45^+^) cell subsets as measured by flow cytometry. A subset (n = 33) of the most relevant WBC population representing innate and adaptive cell compartments was selected. Data were presented as WBC percentages based on the cell count of each subpopulation by its respective population (one level up).

In addition, CCR5 surface expression on monocytes and T lymphocyte subsets were quantified, including naive T cells (CD45RA + CCR7+), central memory T cells (CM, CD45RA-CCR7+), effector memory T cells (EM, CD45RA-CCR7-), effector memory T cells expressing CD45RA (TEMRA, CD45+CCR7-), and the total pool effector T memory cells (TEM, CD45RA−/+CCR7-). Furthermore, within the CD4^+^T cells, CD4^+^ naive regulatory (nTreg, CD45RA+CD25^+^) and CD4^+^ memory regulatory (mTreg, CD45RA-CD25++) cell subsets were identified. The level of CCR5 expression on the cell populations was expressed as the geometric mean of fluorescence intensity (MFI). The level of CCR5 expression in granulocytes was used as an internal negative control. The flow cytometry protocol for identifying CCR5-positive cell subsets was confirmed in three fluorescence minus one (FMO) controls.

### Quantification and statistical analysis

#### Quality control of proteomic data

The proteomic data from the discovery cohort were normalized using inter-plate controls for batch variation correction and were reported in the log_2_ scale. Data values below the limit of detection (<LOD) were handled using the actual measured values to increase the statistical power and give a complete data distribution. Outlier detection was done using principal component analysis (PCA), in which data points falling ≥3 standard deviations (SD) from the mean of principal component one (PC1) and two (PC2) were excluded. Proteins with (1) <LOD values >25% of the samples in both groups (PLHIV and HC) and (2) a difference in the <LOD values between PLHIV and HC <20% were excluded. For analysis within the PLHIV, proteins with <LOD values >25% of the samples were excluded for the follow-up data analysis. Details of pre-analytical steps for both discovery and validation cohort were described in Figure S1. Percentages of <LOD values per protein were visualized in Figure S2.

#### Differential expression protein analysis

For protein analyses in the discovery cohort, we compared the relative concentration of plasma inflammatory proteins between PLHIV and HC. During QC per sample, 12 outliers and one sample with no measurements were excluded from the analysis. In total, 78 proteins in 192 PLHIV and 416 HC participants from the discovery cohort were available for differential expression (DE) analysis (Figure S1).

In the validation cohort, we selected the significantly differentially expressed proteins (DEP) (n = 64) identified in the discovery cohort. Similar pre-analytical approaches were implemented in the data analysis as in the discovery stage. Sixty-two proteins were used for DE analysis after quality control per protein, as described above. After excluding participants that were not using ART (elite controllers; n = 4) and the outliers (n = 10) as described above, we used 649 PLHIV and 98 HC for DE analysis (Figure S1).

We used a linear regression model with age and sex as covariates in the discovery and validation cohort. Adjustment for multiple testing comparisons was done using false discovery rate (FDR) method. Proteins with FDR <0.05 were considered statistically significant in the discovery cohort, and pvalue<0.05 were considered statistically significant in the validation cohort.

#### Clustering analysis

To assess any similarities and dissimilarities within groups, we performed unsupervised hierarchical clustering using k-nearest neighbors with 100 repetitions. Before analysis, NPX values for each protein were scaled to have a mean of 0 and a SD of 1. Results were visualized as a heatmap by calculating the matrix of Euclidean distances from the scaled NPX value. Scaled data exceeding color break value (−2 or 2) were assigned with corresponding maximum or minimum colors.

#### Network analysis of DEP

Network analysis was performed using Spearman’s rank correlation, and association with moderate to high correlation coefficient (rho>3) was visualized. Each protein was visualized as a node and colored based on node’s importance according to node’s closeness. For the dendrogram of DEP between PLHIV and HC, the distances between proteins measured in PLHIV of the discovery cohort were calculated using Ward’s agglomeration method, and hierarchical clustering of the proteins was calculated based on the pairwise distances of proteins using Spearman’s rank correlation.

#### Random forest

Random forest classification model was performed to predict the high and low inflammation groups of PLHIV in the discovery and validation cohort. In addition to demographic factors (age, sex, and BMI), concentration of DEP between the high and low inflammation groups of the discovery (n = 71) and validation cohort (n = 69) were used as input. Before the random forest-based modeling, PLHIV in the discovery and validation cohorts were randomly split into a training (70%) and a validation set (30%). The model was built with 1000 trees and 4 (the discovery cohort) or 2 (the validation cohort) random variables that were considered at each tree. This model was chosen based on the best prediction accuracy on the training set. The importance of each variable in the prediction model was expressed as a mean decrease in accuracy values. The performance of the prediction model of the discovery and validation cohort was visualized using the receiver operating characteristic curve (ROC).

#### Data analysis and visualization

All statistical analyses were performed using R version 4.0.2. The following R packages were used for results visualization: ‘ggbiplot’ for PCA plot, ‘ComplexHeatmap’ for unsupervised hierarchical clustering analysis, ‘randomForest’ for random forest analysis, ‘tidygraph’ and ‘ggraph’ for network analysis, and ‘ggplot2’ for the rest of plots. ‘Limma’ was used for DE analysis which uses an empirical Bayes method to moderate the standard errors of the estimated log-fold changes (Ritchie et al., 2015). ‘randomForest’ and ‘ROCR’ were used for random forest and ROC analyses, respectively. Complementary approach to identify the cellular origin of DEP was performed using single-cell transcriptomic publicly available data from the Human Proteomic Atlas (HPA) project (proteinatlas.org) (Karlsson et al., 2021).

## Data Availability

• All data reported in this article will be shared by the lead contact on request. • This study does not report original code. • Any additional information required to reanalyze the data reported in this article is available from the lead contact on request.

## Supplemental information

Document S1. Figures S1–S 10

Table S1. Differentially expressed proteins (n = 29) between PLHIV and HC in the discovery and validation cohorts, related to Figure 2B Proteins are presented in alphabetical order. Log_2_ fold change indicates the log_2_ difference of protein concentrations in PLHIV compared to HC. P-values were calculated using a linear regression model with age and sex as covariates, whereas FDR indicates the adjusted p-value using Bonferroni correction for multiple testing comparisons.

Table S3. Differentially expressed proteins (n = 67) between low and high inflammation group in the discovery and validation cohort, related to Figure 4E Proteins are presented in alphabetical order. Log_2_ fold change indicates the log_2_ difference of protein concentrations in the high inflammation group compared to the low inflammation group. P-values were calculated using a linear regression model with age and sex as covariates, whereas FDR indicates the adjusted p-value using Bonferroni correction for multiple testing comparisons.
