# Supplementary material for: Targeted plasma proteomics reveals upregulation of distinct inflammatory pathways in people living with HIV
Source: iScience. 2022 Sep 7;25(10):105089. doi: 10.1016/j.isci.2022.105089 (PMC9494231; doi:10.1016/j.isci.2022.105089)
Supplement: Document S1. Figures S1–S — 10 [file mmc1.pdf]

iScience, Volume *25*

Supplemental information

Targeted plasma proteomics reveals upregulation of distinct inflammatory pathways in people living with HIV

Nadira Vadaq, Lisa van de Wijer, Louise E. van Eekeren, Hans Koenen, Quirijn de Mast, Leo A.B. Joosten, Mihai G. Netea, Vasiliki Matzaraki, and André J.A.M. van der Ven

Supplemental information

Supplemental Figures

Figure S1. Quality controls of proteomics data, Related to Figure 1A.

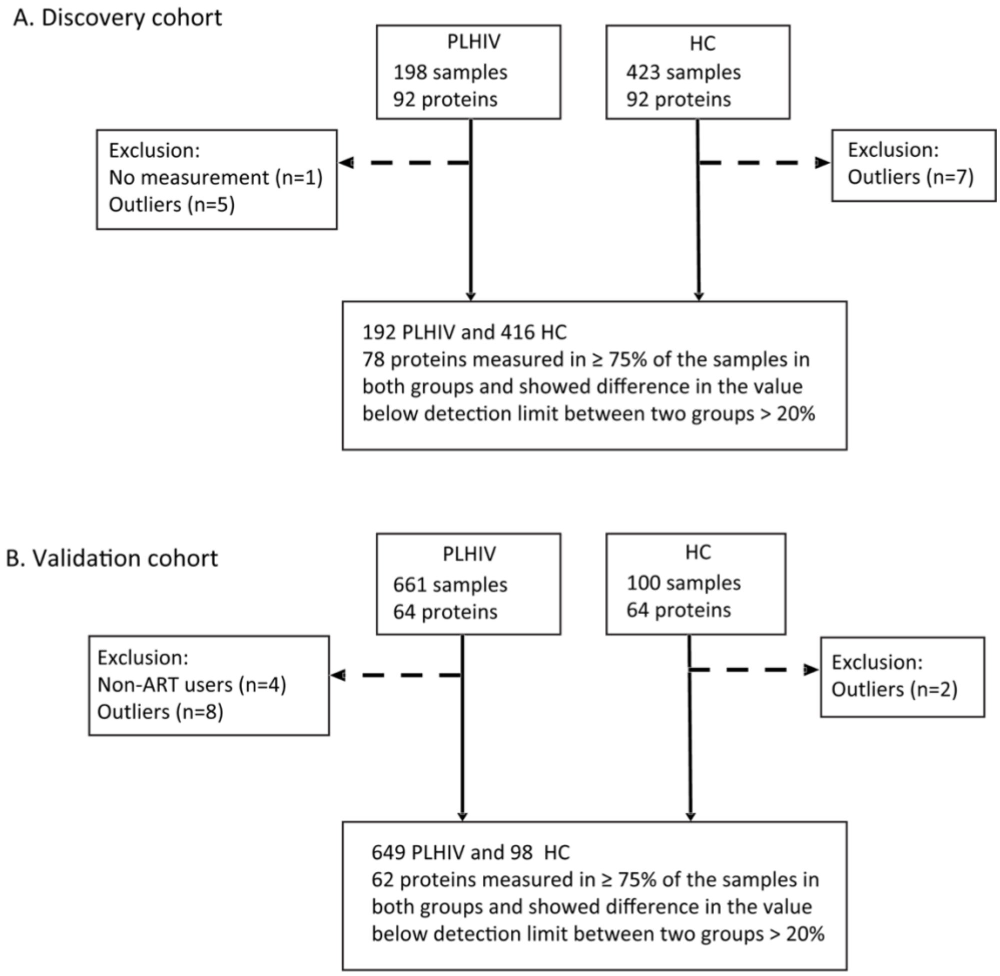

Schematic representation showing quality control (QC) per sample and per protein prior to downstream data analysis in the discovery and validation cohort. **(A)** In the discovery cohort, the initial number of samples with proteomics measurement were 198 people living with HIV (PLHIV) and 423 healthy controls (HC). One sample with no measurement in PLHIV was excluded. In addition, outliers detection were done using principal component analysis (PCA) in which data points falling in ≥3 standard deviations (SD) from the mean of principal component one (PC1) and two (PC2) were excluded. Five and seven outliers were excluded from the PLHIV and HC group respectively. After removing outliers based on the proteomic data, the final number of samples used in the discovery cohort for downstream data analysis were 192 PLHIV and 416 HC. Upon QC per protein, we used proteins that were (1) measured in at least 75% of samples from PLHIV or HC, and (2) showed a difference in the value below the limit of detection (<LOD) between PLHIV vs HC >20%, resulting in a total number of 78 proteins in the discovery cohort. **(B)** In the validation cohort, the initial number of samples with protein measurements were 661 PLHIV and 100 HC. In PLHIV group, participants that were not using antiretroviral therapy (n=4) were excluded from the analysis. In addition, we excluded eight and two outliers as described above from the PLHIV and HC respectively. The final number of samples used in the validation cohort for downstream data analysis were 649 PLHIV and 98 HC. Sixty-four proteins were found to be differentially expressed between PLHIV and HC from the discovery cohort. Therefore, we performed QC per protein for 64 proteins in the validation cohort, of which 62 proteins passed QC as described above.

Figure S2. Protein detectability of study participants, Related to Figure 1A.

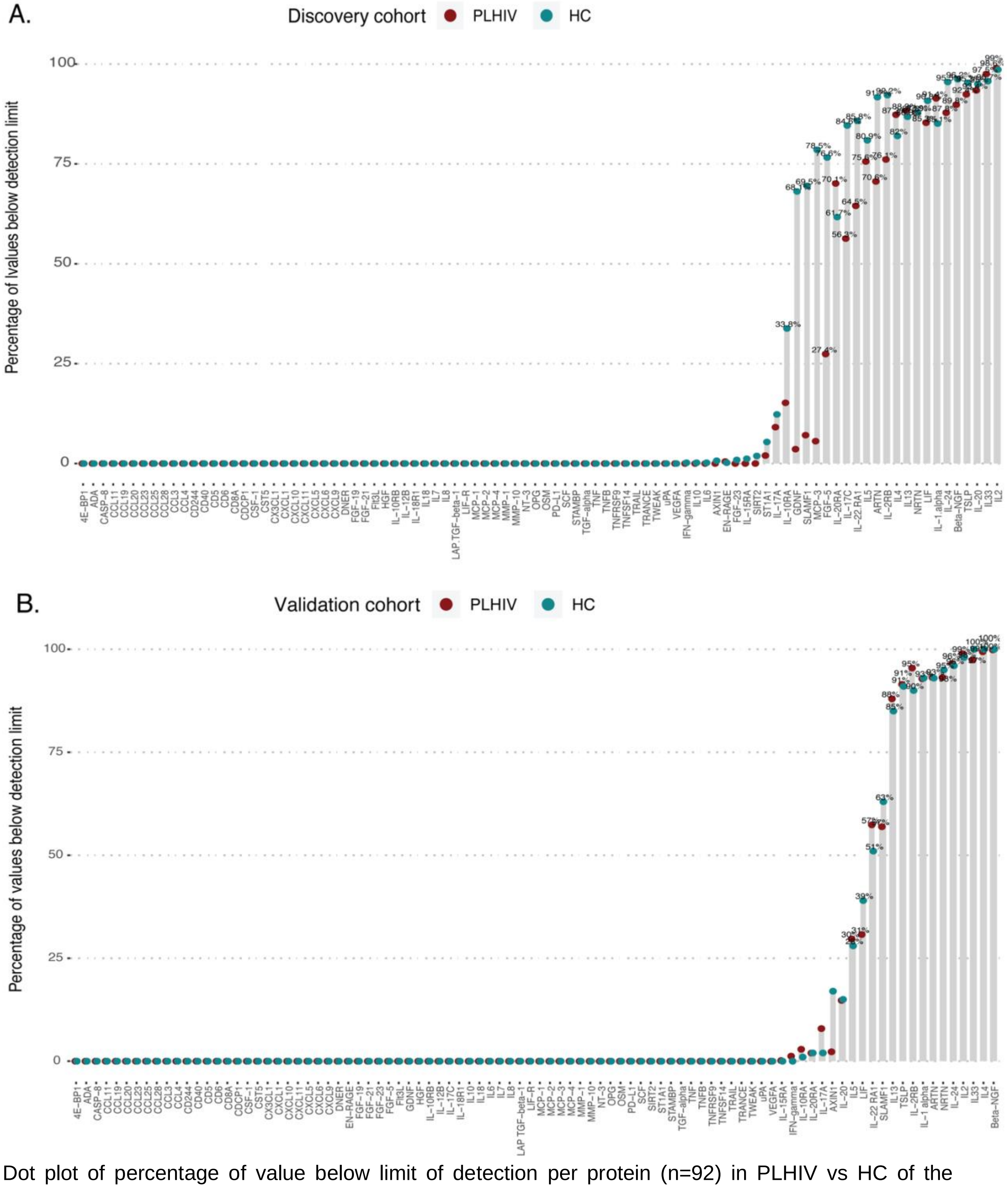

Dot plot of percentage of value below limit of detection per protein (n=92) in PLHIV vs HC of the discovery cohort **(A)** and validation cohort **(B)**.

Figure S3. Intercorrelation of proteomics data in PLHIV and HC, Related to STAR Methods.

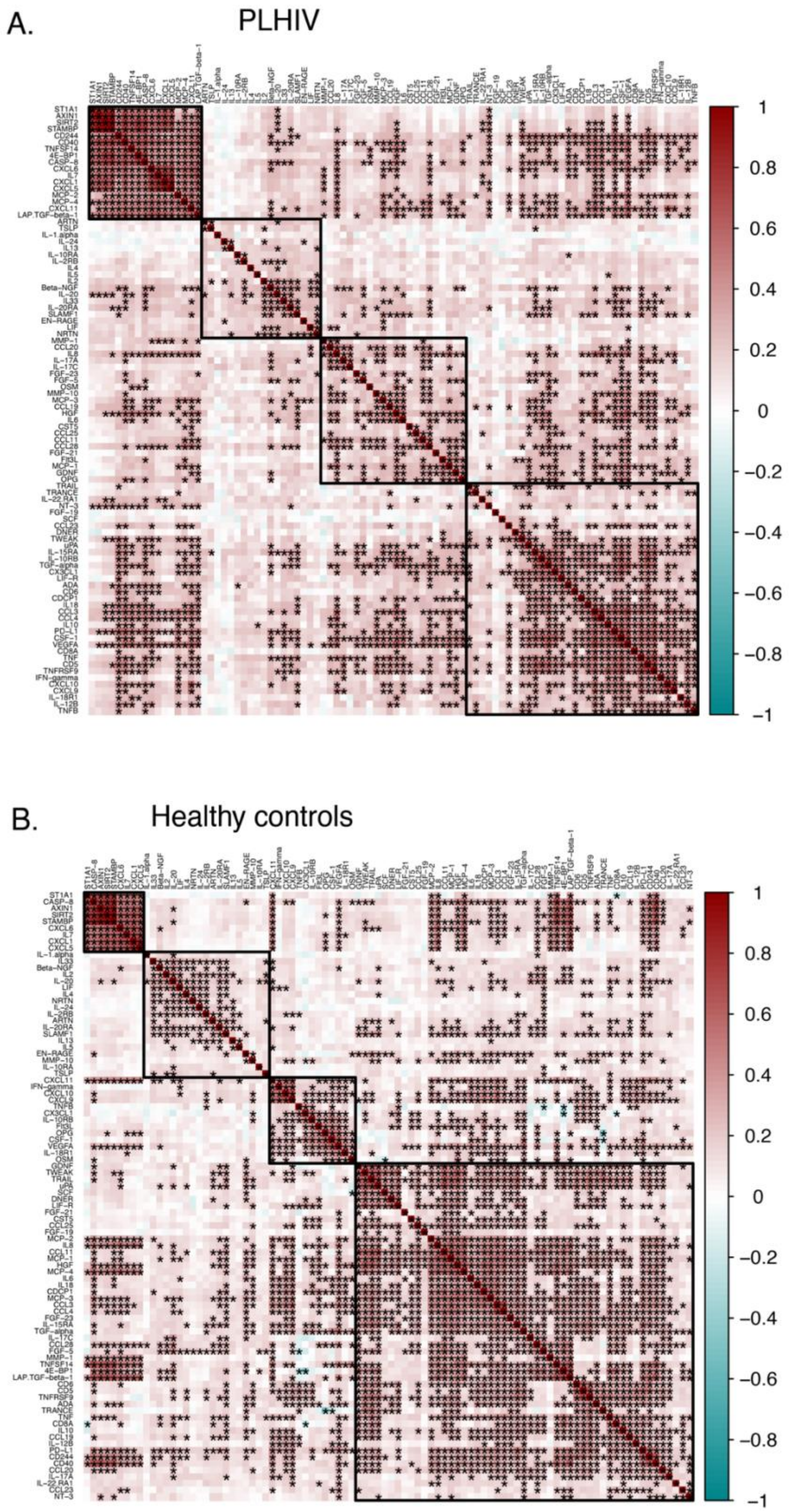

Spearman correlations between plasma inflammatory proteins (n=92) in **(A)** PLHIV (n=192) and **(B)** HC (n=416) from the discovery cohort. The color-coding key depicts coefficient correlation. *FDR P-value<0.0001

Figure S4. Association between plasma inflammatory proteins and cohort charateristics, Related to STAR Methods.

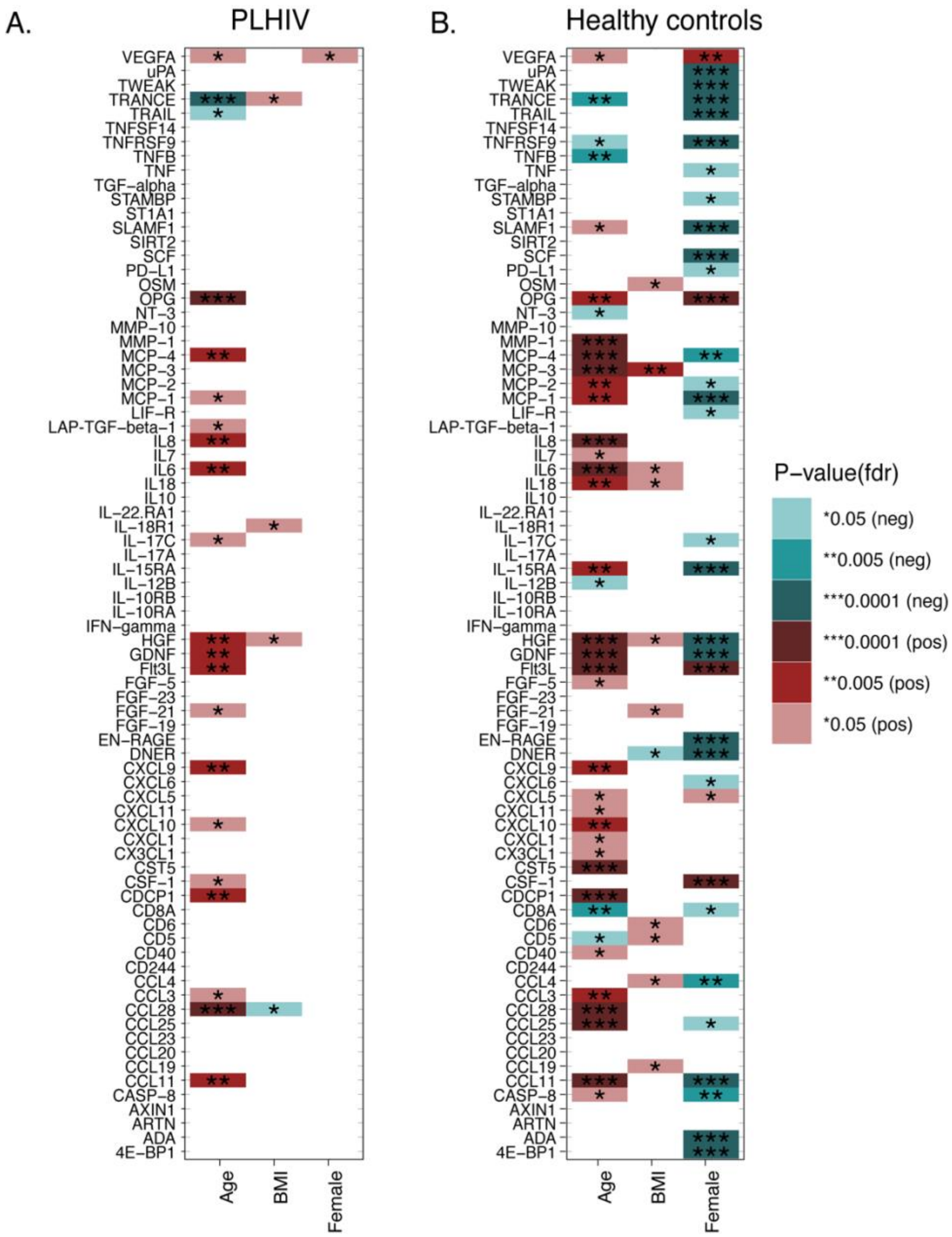

Heat maps showing the p-values (FDR corrected) of correlations between plasma inflammatory proteins (n=78) with age, BMI, and sex in **(A)** PLHIV (n=192) and **(B)** HC (n=416) from the discovery cohort. The color-coding key depicts FDR corrected p-values. The analysis was performed using a linear regression model with adjustment for other metadata.

Figure S5. Differential expression analysis of plasma inflammatory proteins between PLHIV and HC of the discovery cohort, Related to Figure 1D.

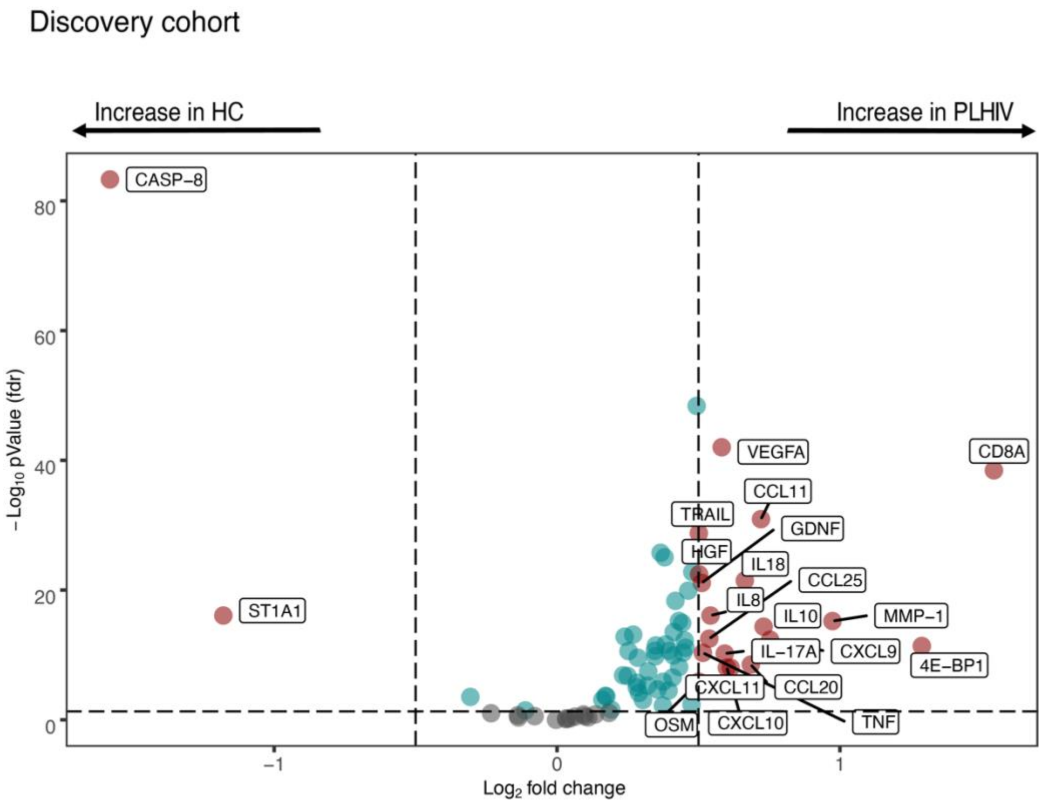

Volcano plot of differential expression of proteins (n=78) between PLHIV (n=192) and HC (n=404) from the discovery cohort. The analysis was performed using a linear regression model with age, sex, BMI, and smoking status as covariates. Fold change in the x-axis refers to the difference in the mean of log2 NPX values between PLHIV and HC. Only proteins that show FDR<0.05 (-log10 p-value > 1.3) and log2 fold change >0.5 were annotated.

Figure S6. Differential expression analysis of plasma inflammatory proteins between PLHIV and HC of the validation cohort, Related to Figure 2A and 2B.

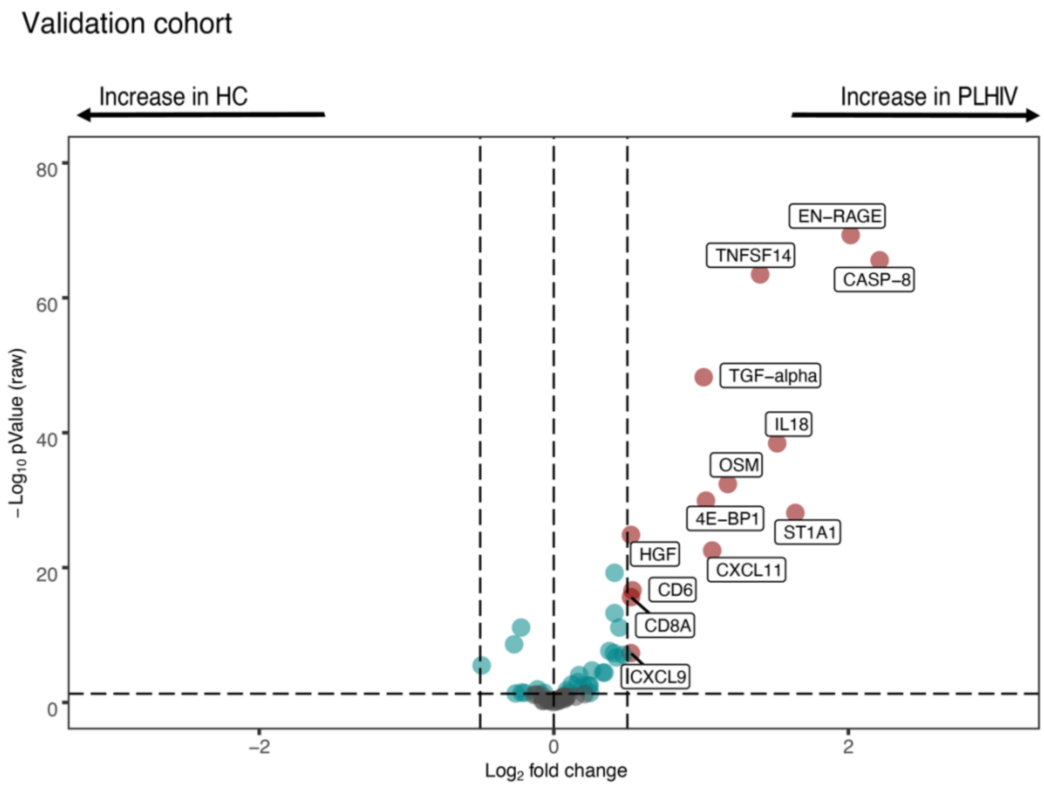

Volcano plot of differential expression of proteins (n=62) between PLHIV (n=649) and HC (n=98) from the validation cohort. The analysis was performed using a linear regression model with age and sex as covariates. Fold change in the x-axis refers to the difference in the mean of log2 NPX values between PLHIV and HC. Only proteins that show FDR<0.05 (-log10 p-value > 1.3) and log2 fold change >0.5 were annotated.

Figure S7. Cellular origin of differentially expressed proteins, Related to Figure 2B and Table S1.

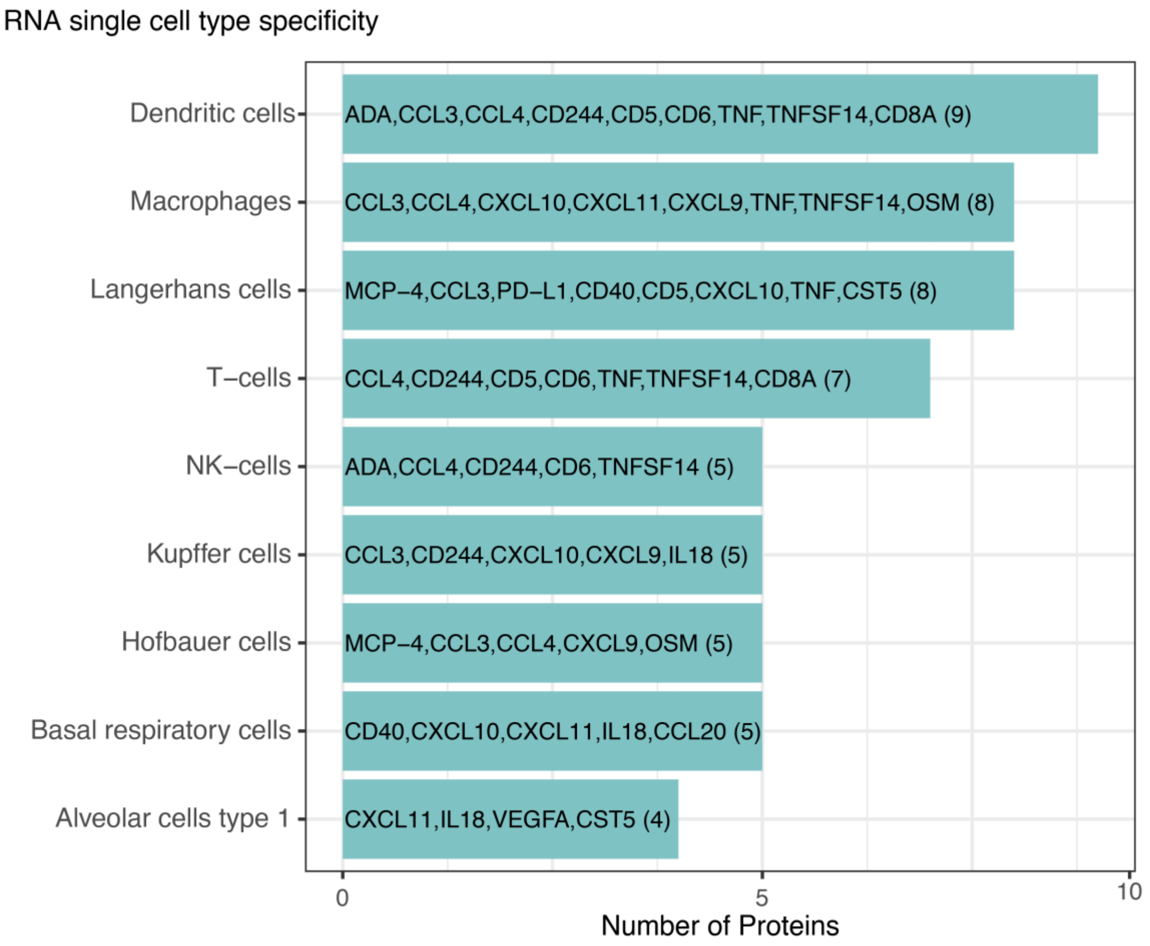

Barplot showing the number of the cellular origin of differentially expressed proteins (n=29) according to single-cell transcriptomic publicly available data from the Human Proteomic Atlas (HPA) project (proteinatlas.org).

Figure S8. Comparison of HIV clinical parameters between PLHIV with high and low inflammation group, Related to Figure 4D.

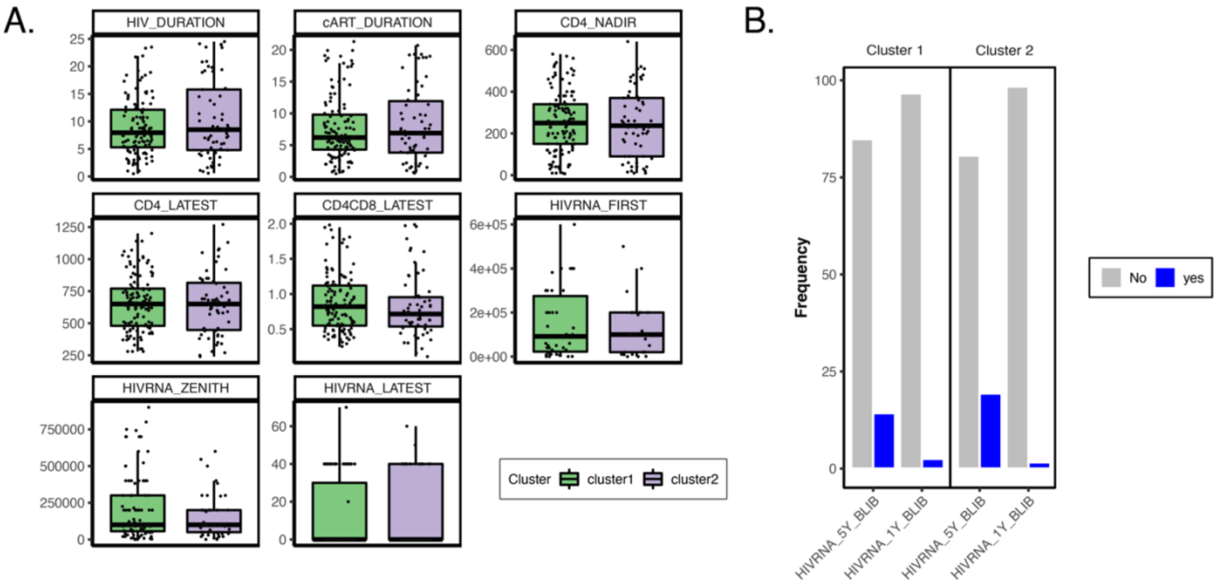

A. Boxplot showing the comparison of CD4 nadir and latest, CD4/CD8 ratio, HIV RNA zenith and latest value, HIV and ART duration between PLHIV on high and low inflammation group of the discovery cohort (all p-value>0.05). The analysis was performed by linear regression model using age and sex as covariates. In all box plots, the in-box line defines the median value, hinges depict 25th and 75th percentiles and whiskers extend to ±1.5 interquartile ranges; each dot indicates an individual participant. **B.** Barplot showing the comparison of viral blips frequency between PLHIV on high and low inflammation group of the discovery cohort (Chi-square p-value>0.05).

Figure S9. Clustering analysis of plasma inflammatory profiles in PLHIV of validation cohort, Related to Figure 4A and 4E.

Validation cohort

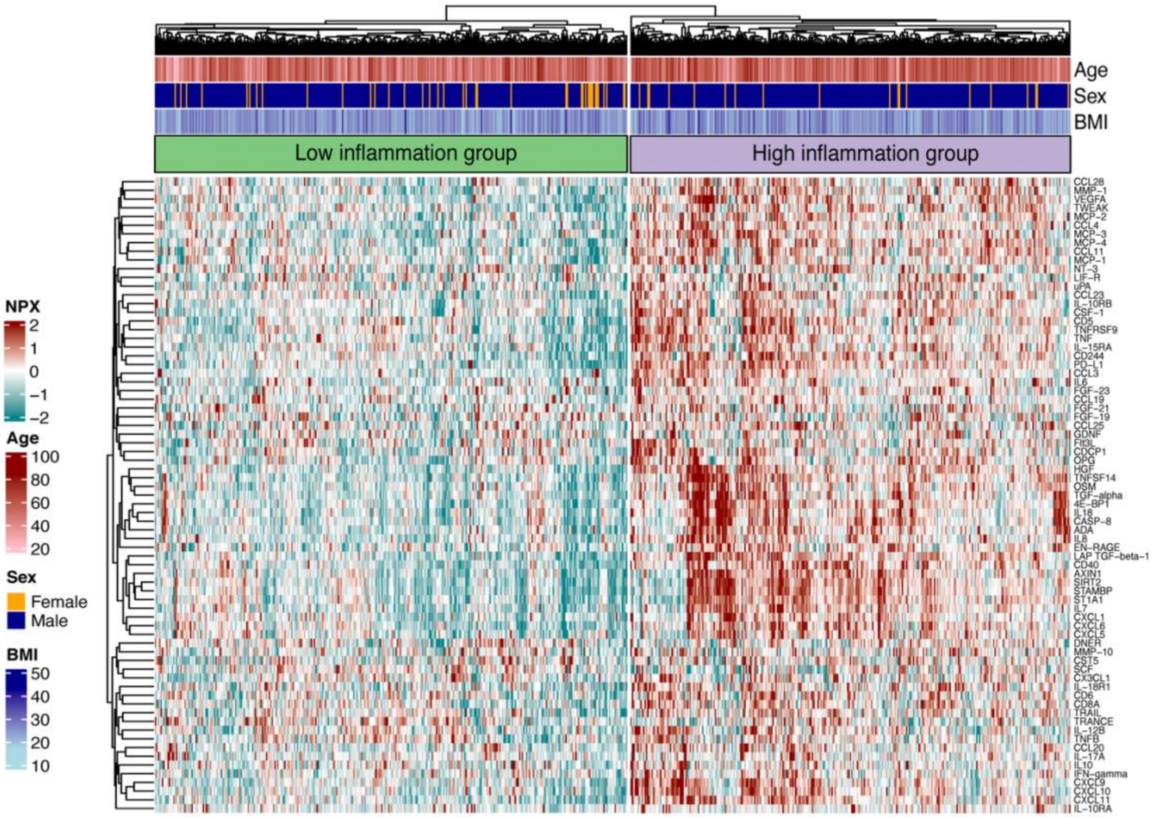

Unsupervised k-means clustering of PLHIV using the relative concentration of plasma inflammatory proteins (n=72) from the validation cohort. Green and purple color represent individuals belong to the low (n=336) and the high inflammation group (n=313), respectively. Data are shown as scaled log_2 NPX values. The color code indicates the relative concentration of proteins across the samples of the two clusters. Red and green colors indicate high and low protein concentrations respectively. Age, sex, BMI, are presented in color-coded scale.

Figure S10. Relation between plasma inflammatory proteins and HIV-related parameters, Related to Table 1.

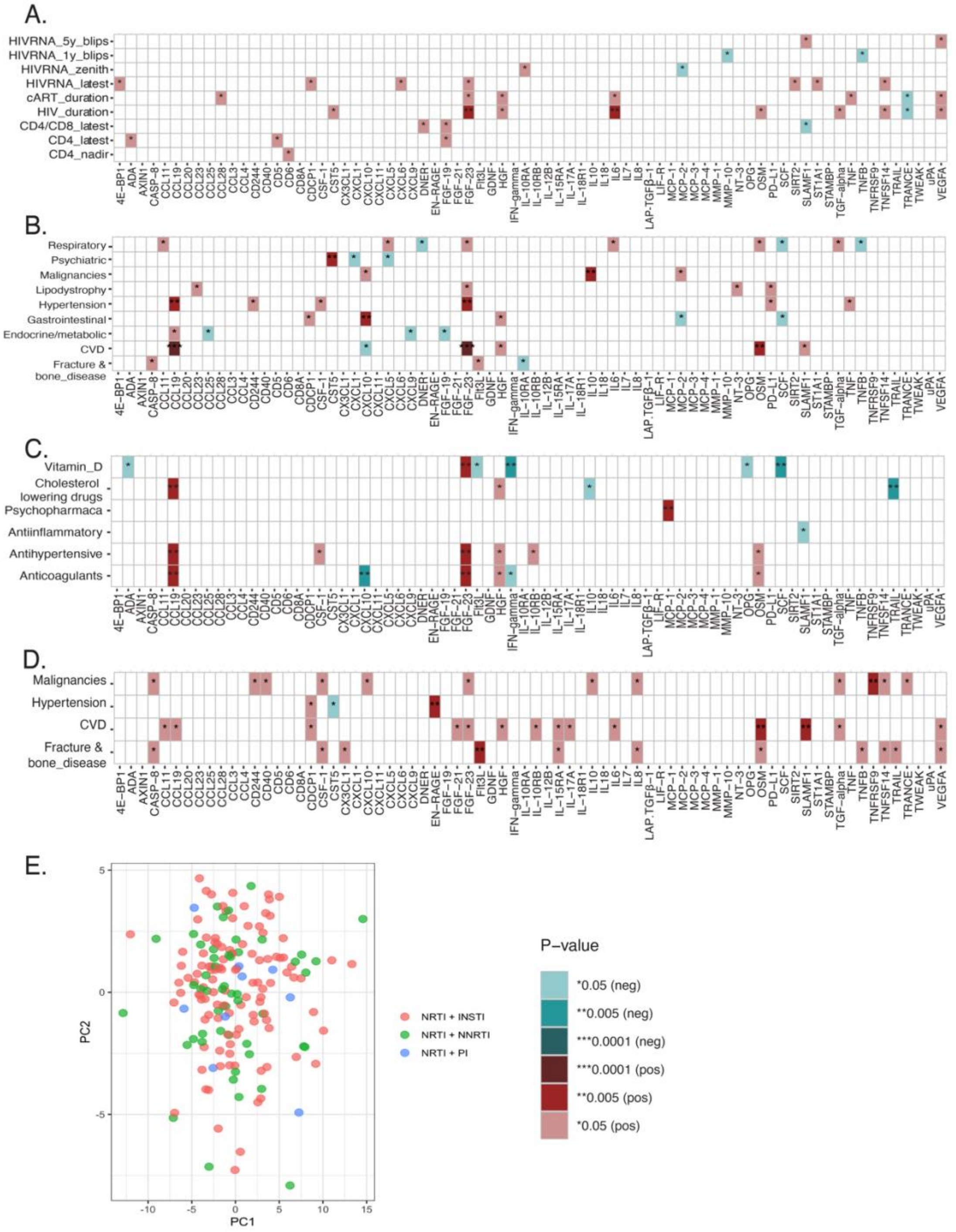

Heatmaps showing the correlations between plasma inflammatory proteins (n=78) with **(A)** HIV clinical parameters, **(B)** comorbidities at baseline, **(C)** co-medication at baseline, **(D)** 5-year follow-up clinical events. **(E)** PCA of plasma inflammatory proteins (n=78) in people with HIV using different classes of anti-retroviral therapy: combination of NRTI and INSTI (n=111); NRTI and NNRTI (n=49); NRTI and PI (n=9).

Table S2. Details of malignancy and cardiovascular disease during 5-year follow up in the high and low inflammation group of PLHIV from discovery cohort, Related to Figure 4D.

Low inflammation group (n=123)		High inflammation group (n=65)	
**Type of malignancy**	**Frequency (n/N)**	**Type of malignancy**	**Frequency (n/N)**
Hodgkin lymphoma	1/123	Non-Hodgkin lymphoma	1/65
Anal dysplasia	2/123	Anal cancer	1/65
Prostate cancer	1/123	Kaposi sarcoma	1/65
Skin cancer	1/123	Skin cancer	2/65
		Esophageal cancer	1/65
		Lung cancer	1/65
		Rectal cancer	1/65
		Larynx cancer	1/65

Low inflammation group (n=123)		High inflammation group (n=65)	
**Type of cardiovascular disease**	**Frequency (n/N)**	**Type of cardiovascular disease**	**Frequency (n/N)**
Heart failure	3/123	Heart failure	2/65
Myocardial infarct	3/123	Myocardial infarct	2/65
Cerebrovascular accident	1/123	Cerebrovascular accident	2/65
Angina pectoris	1/123	Angina pectoris	2/65
Transient ischemic attack	1/123		
